# MIF/CD74 axis is a target for novel therapies in colon carcinomatosis

**DOI:** 10.1186/s13046-016-0475-z

**Published:** 2017-01-23

**Authors:** Fabio Bozzi, Angela Mogavero, Luca Varinelli, Antonino Belfiore, Giacomo Manenti, Claudio Caccia, Chiara C. Volpi, Galina V. Beznoussenko, Massimo Milione, Valerio Leoni, Annunziata Gloghini, Alexandre A. Mironov, Ermanno Leo, Silvana Pilotti, Marco A. Pierotti, Italia Bongarzone, Manuela Gariboldi

**Affiliations:** 1 Department of Diagnostic Pathology and Laboratory Medicine, Fondazione IRCCS Istituto Nazionale dei Tumori, via G. Venezian 1, Milan, 20133 Italy; 2 Department of Experimental Oncology and Molecular Medicine, Fondazione IRCCS Istituto Nazionale dei Tumori, via G. Amadeo 42, Milan, 20133 Italy; 3 Molecular Genetics of Cancer, Fondazione Istituto FIRC di Oncologia Molecolare, via Adamello 16, Milan, 20139 Italy; 4 Proteomics Laboratory Department of Experimental Oncology and Molecular Medicine, Fondazione IRCCS Istituto Nazionale dei Tumori, via G. Amadeo 42, Milan, 20133 Italy; 5 Department of Predictive and Preventive Medicine, Fondazione IRCCS Istituto Nazionale dei Tumori, via G. Amadeo 42, Milan, 20133 Italy; 6 Laboratory of Clinical Pathology and Medical Genetics, Fondazione IRCCS ‘Carlo Besta’ Istituto Neurologico, via G. Amadeo 42, Milan, 20133 Italy; 7 Fondazione Istituto FIRC di Oncologia Molecolare, via Adamello 16, Milan, 20139 Italy; 8 Colorectal Cancer Unit-Department of Surgery, Fondazione IRCCS Istituto Nazionale dei Tumori, via G. Venezian 1, Milan, 20133 Italy

**Keywords:** Organoids, Metabolism, AMPK, 4-IPP, Macrophage migration inhibitory factor, Metformin

## Abstract

**Background:**

Strategies aimed at obtaining a complete cytoreduction are needed to improve long-term survival for patients with colorectal cancer peritoneal carcinomatosis (CRC-pc).

**Methods:**

We established organoid models from peritoneal metastases of two naïve CRC patients. A standard paraffin inclusion was conducted to compare their 3D structure and immunohistochemical profile with that of the corresponding surgical samples. RNA expression levels of the CRC stem cell marker LGR5 was measured by in situ hybridization. The secretome of organoids was profiled by mass spectrometry. Energy homeostasis of organoids was interfered with 4-IPP and metformin. Biochemical and metabolic changes after drug treatments were investigated by western blot and mass spectrometry. Mitochondria impairment was evaluated by electron microscopy and mitotraker staining.

**Results:**

The two organoids recapitulated their corresponding clinical samples in terms of 3D structure and immmunoistochemical profile and were positive for the cancer stem cells marker LGR5. Proteomic analyses of organoids highlighted their strong dependence on energy producing pathways, which suggest that their targeting could be an effective therapeutic approach.

To test this hypothesis, we treated organoids with two drugs that target metabolism acting on AMP-activated protein kinase (AMPK), the main regulator of cellular energy homeostasis, which may act as metabolic tumour suppressor in CRC. Organoids were treated with 4-IPP, an inhibitor of MIF/CD74 signalling axis which activates AMPK function, or metformin that inhibits mitochondrial respiratory chain complex I.

As a new finding we observed that treatment with 4-IPP downregulated AMPK signalling activity, reduced AKT phosphorylation and activated a JNK-mediated stress-signalling response, thus generating mitochondrial impairment and cell death. Metformin treatment enhanced AMPK activation, decreasing the activity of the anabolic factors ribosomal protein S6 and p4EBP-1 and inducing mitochondrial depolarization.

**Conclusion:**

We provide evidence that the modulation of AMPK activity may be a strategy for targeting metabolism of CRC-pc organoids.

**Electronic supplementary material:**

The online version of this article (doi:10.1186/s13046-016-0475-z) contains supplementary material, which is available to authorized users.

## Background

Intraperitoneal dissemination is a common progression feature for colorectal cancer (CRC). For the past two decades, aggressive treatments of CRC peritoneal carcinomatosis (CRC-pc), such as cytoreductive surgery plus hyperthermic intraperitoneal chemotherapy, has improved long-term survival [1]. However, the majority of cases present widespread metastases which cannot be destroyed by systemic chemotherapy; therefore, the development of new strategies to prevent the progression of metastatic disease is imperative.

Tumour-initiating, or cancer stem cells (CSCs), have a key role in metastatic CRC (hepatic and/or lung) and chemotherapy resistance [2]; however, limited information is known about the role of CSCs in CRC-pc development. The first step towards the identification of new therapeutic alternatives requires the development of dedicated pre-clinical models that in vitro and in vivo recapitulate the biological features of CRC-pc.

Past years have seen unprecedented developments of three-dimensional (3D) cultures called cancer organoids. A CRC organoid is obtained by allowing cells (derived from a primary tumour specimen) to self-organize into a 3D structure that recapitulates the original glandular organization commonly observed in human CRC surgical samples. Organoids grow as irregular compact structures, and can be expanded indefinitely and represent the physiology of native tumours much better than traditional cell lines [3]. We have established (in serum-free medium) two CRC-pc organoid cultures of peritoneal metastatic lesions from two CRC patients that showed enrichment in the expression of the CRC CSC marker leucine-rich repeat containing G protein-coupled receptor 5 (LGR5) a member of the canonical WNT pathway and a well-recognized marker of the cell progenitor population located at the crypt-base [4, 5].

In this study, we hypothesized that CRC organoids may secrete a variety of growth factors, cytokines, proangiogenic factors, exosomes, and even extracellular matrix components, some of which are fundamental for maintaining self-renewal and proliferative capacity. Using the advantageous properties of “serum-free” growth conditions for both organoids, we searched for factors with potential therapeutic relevance in their “secretome” compartment produced during cell culture [6]. We found the prominent expression of metabolic pathways mainly related to oxidative homeostasis and glucose metabolism and focused on the macrophage migration inhibitory factor (MIF)/CD74/mitochondria axis, and on the relative 4-iodo-6-phenylpyrimidine (4-IPP) inhibitor, as playing an important role in JNK modulation of the cellular response to reactive oxygen species [7]. To further examine the importance of targeting metabolism in organoids, we studied the effects of direct perturbation of mitochondrial integrity using metformin, which inhibits respiratory-chain complex I, thus inducing a pro-cell death decline in energy status.

## Methods

### Cell culture and tissue samples

Two cancer organoids (CRC-pc1 and CRC-pc2, hereinafter referred to as C1 and C2) were established from peritoneal metastases of naïve patients with stage IV, grade 2 (C1) and grade 3 (C2) CRC following protocols and conditions published by Sato T et al. [8]. BRAF and KRAS mutation patterns in organoids reflected those in the original tumours. Formalin fixed paraffin embedded (FFPE) tissue samples obtained from additional CRC-pc patients (C3, C4 and C5) were included in the study for validation experiments.

The two organoids were maintained in F-12 medium (DMEM F12, Gibco, Carlsbad, CA, USA) as previously described [8].

This study was approved by the Institutional Review Board of Fondazione IRCCS Istituto Nazionale dei Tumori and each patient provided written informed consent to donate remaining tissues after diagnostic procedures.

### Morphology and immunohistochemistry

A standard paraffin inclusion of both organoids was conducted to compare their 3D structure and immunohistochemical profile with those of the corresponding surgical samples. Immunohistochemistry was done with 3-μm FFPE tissue sections. The antibodies used for immunohistochemistry analyses are listed in Additional file 1: Table S1.

### In Situ Hybridization (ISH)

ISH for LGR5 RNA expression was manually performed using the RNAscope 2.0 High Definition detection kit (Brown) (Advanced Cell Diagnostics, Inc., Hayward, CA) assay according to the manufacturer’s instructions. Briefly, 5 μm FFPE tissue sections were incubated with Pretreat buffers 1, 2 and with Pretreat Buffer 3 at 40 °C. Slides were hybridized with probes of interest for 2 h at 40 °C, followed by incubations with amplifier reagents 1–6, color development with 3, 3′- diaminobenzine (DAB) and counterstaining with haemotoxylin. RNAscope probes used were LGR5 (311021), the negative control bacterial DapB (310043) and endogenous UBC (310041), used as positive control. Positive staining signals were identified as brown punctuate dots present in the cytoplasm and/or nucleus [9].

### Nano-scale liquid chromatographic tandem mass spectrometry (nLC-MS/MS) analysis

Proteins secreted in the medium (secretome) by C2 organoids were collected and the secretome was profiled as previously described [10].

### Statistical and GO (Gene Ontology) analyses

The Search Tool for the Retrieval of INteracting Genes/proteins (STRING) database [11] (9.1 version (Database issue):D412-416) was used for prediction of protein subcellular localization. Resulting proteins were also analysed using PANTHER (Protein ANalysis THrough Evolutionary Relationships) system (http://www.pantherdb.org) [12]. Network analyses were performed using the Ingenuity Pathways Analysis (Ingenuity Systems) software.

### Sample preparation, SDS-PAGE and immunoblotting

Organoids were cultured for 18 h, and exposed to drugs for the times indicated. Cell pellets were solubilized as previously described [13]. Protein concentrations, SDS-PAGE and electroblotting were determined and performed as previously described [14]. The antibodies used for western blotting analyses are listed in Additional file 1: Table S2.

### Trypan blue exclusion

A standard trypan blue exclusion assay was used to evaluate cell viability after 4-IPP and metformin treatments. Data were analyzed using Student’s *t*-test.

### Drug treatments

For cell treatments, 88.5 mM of 4-iodo-6-phenylpyrimidine (4-IPP) (Calbiochem, Milan, Italy) and 1 M metformin (Sigma-Aldrich, St. Louis, MO, USA) in 100% DMSO and in PBS 1×, respectively, were diluted directly in the cell culture medium to achieve the working concentrations. The final solvent and vehicle concentrations were less than 0.1% for all samples, including controls.

### Isotope dilution mass spectrometry analysis

C2 organoids were treated with 50 or 100 μM 4-IPP and 5 mM metformin. After 24 h, treated cells were lysed and the lysates were analysed by isotope dilution mass spectrometry, as previously described [15].

### Reactive oxygen species detection

C2 organoids were seeded into a 96-well plate. After 24 h, cells were treated with 5 mM metformin for 120 h and with 100 μM 4-IPP for 6 h. Extracellular H_2_O_2_ formation was detected and quantified using the ROS-Glo H_2_O_2_ assay (Promega, Madison, WI, USA) according to instructions provided by the manufacturer. Luminescence intensity was quantified using a microplate reader (Infinite 200, TECAN Group, Ltd., Männedorf, Switzerland).

### Mitochondrial membrane potential analysis

Mitochondrial membrane potential of cells was detected using a MitoProbe™ JC-1 Assay Kit for flow cytometry (Thermo Fisher Scientific Corporation, Carlsbad, CA, USA) after treatment with drugs for the times indicated. Flow cytometry analyses were performed as previously described [16].

### Electron microscopy

Cells were fixed by addition of 2.5% glutaraldehyde in 0.1 M cacodylate buffer (pH 7.2) for 2 h [17]. Next, samples were post-fixed with reduced OsO4 for 1 h [18]. Osmium staining was treated with 0.3% thiocarbohydrazide in O.2 M cacodylate buffer (pH 6.9) for 30 min [19]. Finally, cells were treated with the reduced OsO4 for 1 h in the darkness and placed 50% ethanol, which was gradually replaced by 70, 90 and 100% ethanol. After this dehydratation, samples were embedded in Epon [19]. After 24-h polymerization at 60 °C, samples were cut using Leica FC6 ultramicrotome (Leica, Wien, Austria) and examined in Tecnai 20 transmission electron microscope (FEI, Endhoven, The Netherland) [20].

### Mitotracker staining and confocal microscopy

C2 cells were plated in 100 mm Petri dishes (2 × 10^6^ cells/well), incubated for 18 h before treatment with 50 or 100 μM 4-IPP (24 h) or with 5 mM metformin (120 h). The cells were stained with MitoTracker Deep Red FM (Molecular Probes, Invitrogen, Carlsbad, CA, USA) at a final concentration of 60 nM, then fixed with 4% paraformaldehyde at 37 °C for 15 min, and washed with phosphate-buffered saline. Immunofluorescence staining was performed on 2-μm paraffin-embedded sections, deparaffinised in xylene and rehydrated in graded alcohols. The slides were washed in 0.05 M phosphate-buffered saline, incubated with DAPI 1:35,000 (Molecular Probes) for 10 min, washed again in 0.05 M phosphate-buffered saline and then fixed using Prolong Antifade (Molecular Probes). Confocal laser scanning microscopy was performed using the Leica TCS SP8 X microscope (Leica Microsystems GmbH, Mannheim, Germany).

## Results

### 3D structures, IHC profile and LGR5 expression of the organoids

In-phase contrast microscopy showed both tubular-branched and more round shaped 3D structures present in C1, while C2 was mainly composed of round-shaped cellular aggregates (Additional file 2: Figure S1).

**Fig. 1:**
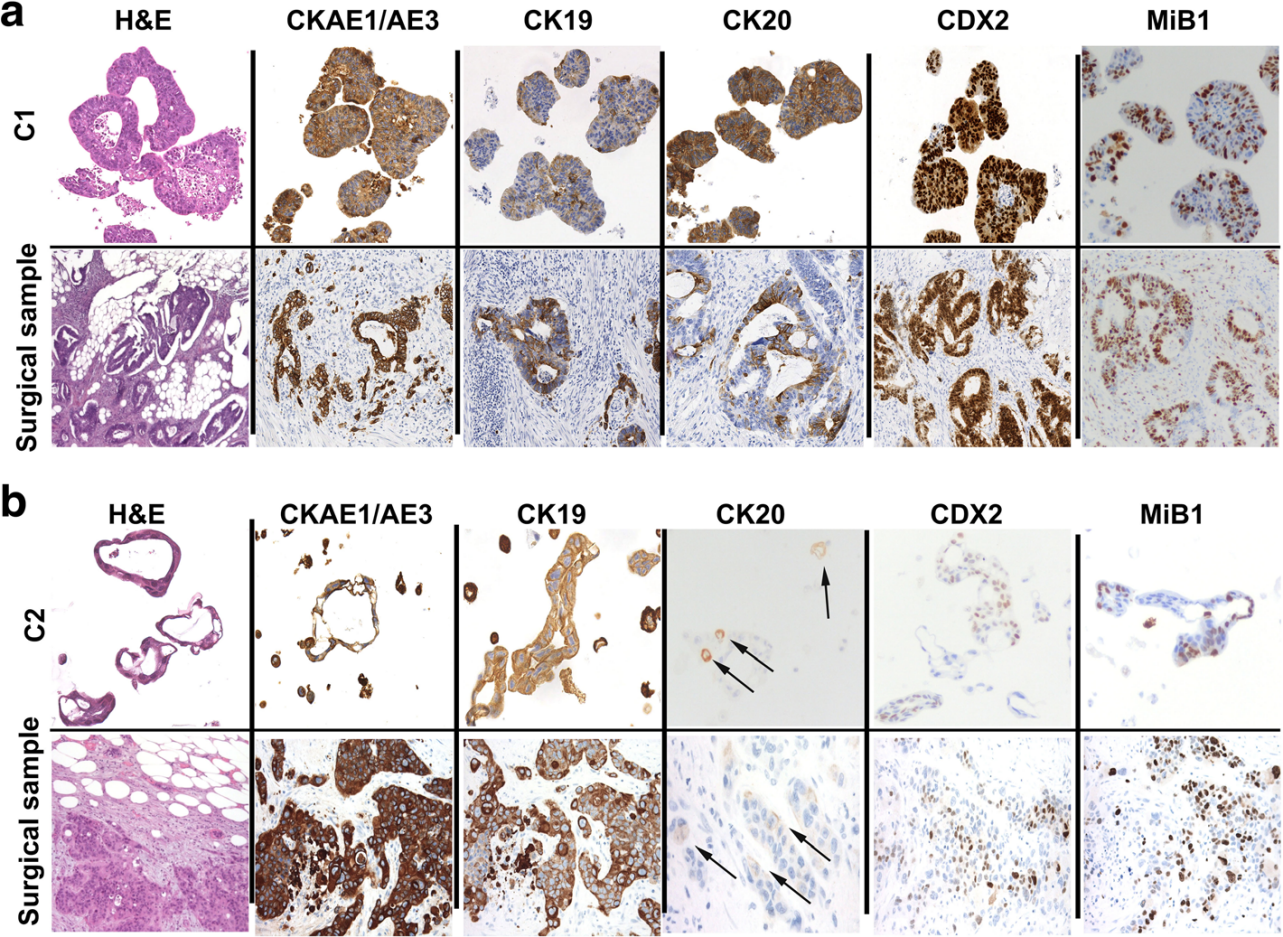
Immunohistochemical profile of FFPE sections from C1 and C2 organoids and corresponding surgical samples. Haematoxylin and eosin (H&E) staining and CK AE1/AE3, CK19, CK20, CDX2 and Ki67 immunostaining of C1 **a** and C2 **b**. Arrows indicate the weak CK20 staining of C2 organoids and of the corresponding surgical sample. Magnifications at 20X

After paraffin inclusion, C1 organoids exhibited glandular structures lined by pseudostratified columnar cells with central fibrinoid core and dirty necrosis along with roundish solid aggregates of not aligned tumour cells. These features mirrored those present in the corresponding histologic sample (Fig. 1a). In C2 organoids the cells exhibited adhesion loss and were organized in ring and/or ribbon-like structures. The corresponding sample showed solid nests of poorly differentiated cells with eosinophilic cytoplasm surrounded by desmoplastic stroma infiltrating omental fat (Fig. 1b). Both organoids expressed Cytokeratins (CK) A1-A3, −19, −20 (Fig. 1) and Claudin 4, and were negative for CK7 and Paired Box 8 (PAX8) (data not shown). C1 retained a high expression of Caudal Type Homeobox 2 (CDX2) compared to C2. Finally, according to the observed rapid growth, both organoids had a very high Ki-67 staining (Mib1) (Fig. 1).

**Fig. 2:**
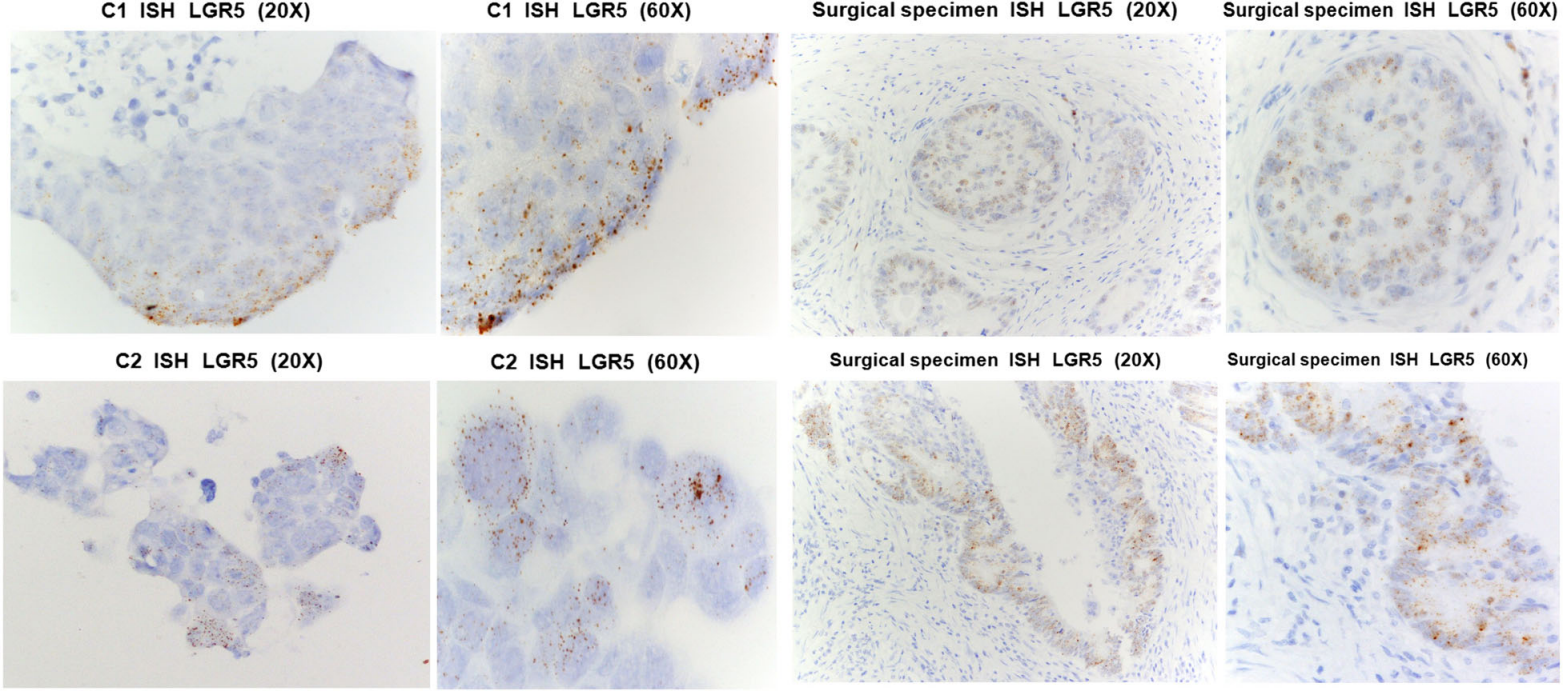
In situ hybridization. in situ hybridization of LGR5 on FFPE sections of C1 and C2 organoids, and of the surgical specimens from which they originated

By ISH, we investigated the expression of LRG5, the marker of the cell progenitor population located at the crypt-base [4, 5]. LRG5 RNA expression was observed in both organoids (Fig. 2). C1 displayed a gradient of LRG5 staining that was stronger in the peripheral sheet of epithelial cells compared to more luminal sheets. Conversely, C2 LGR5 was very focally expressed by some apical and peripheral organoid cells, resulting in a more heterogeneus staining. Transcription of LGR5 was also confirmed in their corresponding clinical FFPE materials (Fig. 2).

Taking advantage from the serum-free growth conditions of CRC-pc organoids, we profiled the secretome compartment to search for novel molecular candidates for therapy involved in CRC-pc development and coupled it with a classical pharmacological approach aimed at exploring the possible implications of these candidates in CRC-pc. Indeed, we also used C1 organoids for validating the results obtained from the use of C2.

We stated that these proteins playing a role in crucial biological processes were secreted by organoids and that they were present also in clinical samples. These studies support the concept that CRC-pc organoids may constitute a pre-clinical model for investigating the biology of CRC-derived peritoneal lesions.

### Over-represented biological processes identified by secretome in C2 organoids

**Fig. 3:**
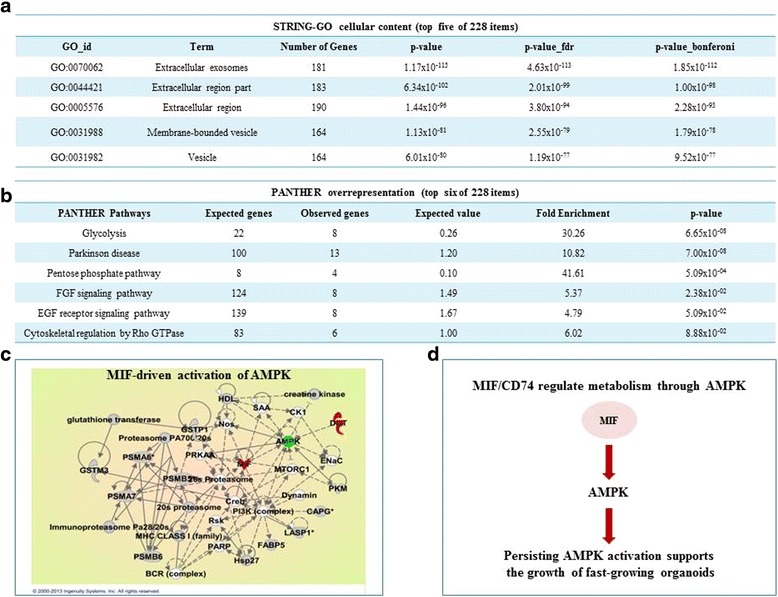
Over-represented Gene Ontologies and pathways in the secretome of C2 organoids. **a** Gene Ontology analysis of secreted proteins. The Search Tool for the Retrieval of INteracting Genes/proteins (STRING) database was used for prediction of protein subcellular localization. STRING confidence score represented the probability of finding functional modules that were selected if the significance (*p*-value) of GO enrichment was less than 0.01. Statistical significance was ascertained with a false discovery rate (FDR) threshold of <0.05. **b** PANTHER overrepresentation test. Proteins involved in metabolic process and regulation of extracellular activities were also major components of the C2 secretome. The table shows the most representative identified overrepresented categories of proteins involved in Parkinson disease, glycolysis and pentose phosphate pathways. Experiments were performed in duplicate. Data were considerate significant when *p* ≤ 0.05. **c** Network analysis was performed using Ingenuity Pathway Analysis software. The analysis shows the connection between the MIF, DDT (MIF-2) with AMPK embedded within the network. The network predicts the potential value and validity of the MIF-AMPK connection. **d** Summary of the modulation of AMPK activity on organoids through MIF

The secretome composition of C2 organoids was investigated using a classical bottom-up proteomic analysis with LC-Orbitrap mass spectrometry. Of the 229 items, 191 (83%) were recognized as proteins with GO extracellular space by STRING (Fig. 3a). PANTHER assessed overrepresentation of proteins involved in Parkinson disease, glycolysis and pentose phosphate pathways (Fig. 3b). These results are consistent with the previous observation that functions related to glucose metabolism and mitochondria homeostasis are downregulated in neurodegeneration, including Parkinson’s disease (PD), but upregulated in colorectal cancer [21].

Analysis of the secreted proteins with annotations in the IPA database identified networks that were enriched in proteins involved in metabolism homeostasis, protein ubiquitination, cell cycle and DNA repair, and were significantly over-represented in cancer (Additional file 2: Figure S2).

### Macrophage migration inhibitory factor (MIF)/CD74 signalling axis

Secretome composition of C2 organoids included proteins potentially able to mediate high metabolic demand, adaptive homeostasis along with enhancing stress resistance. One of them was the multifunctional cytokine MIF (Fig. 3c), the ligand of CD74 receptor. MIF/CD74 axis can promote cell survival by modulating glucose uptake and metabolism through the activation of AKT and AMPK proteins and is considered a potential target for therapy [22] (Fig. 3c).

**Fig. 4:**
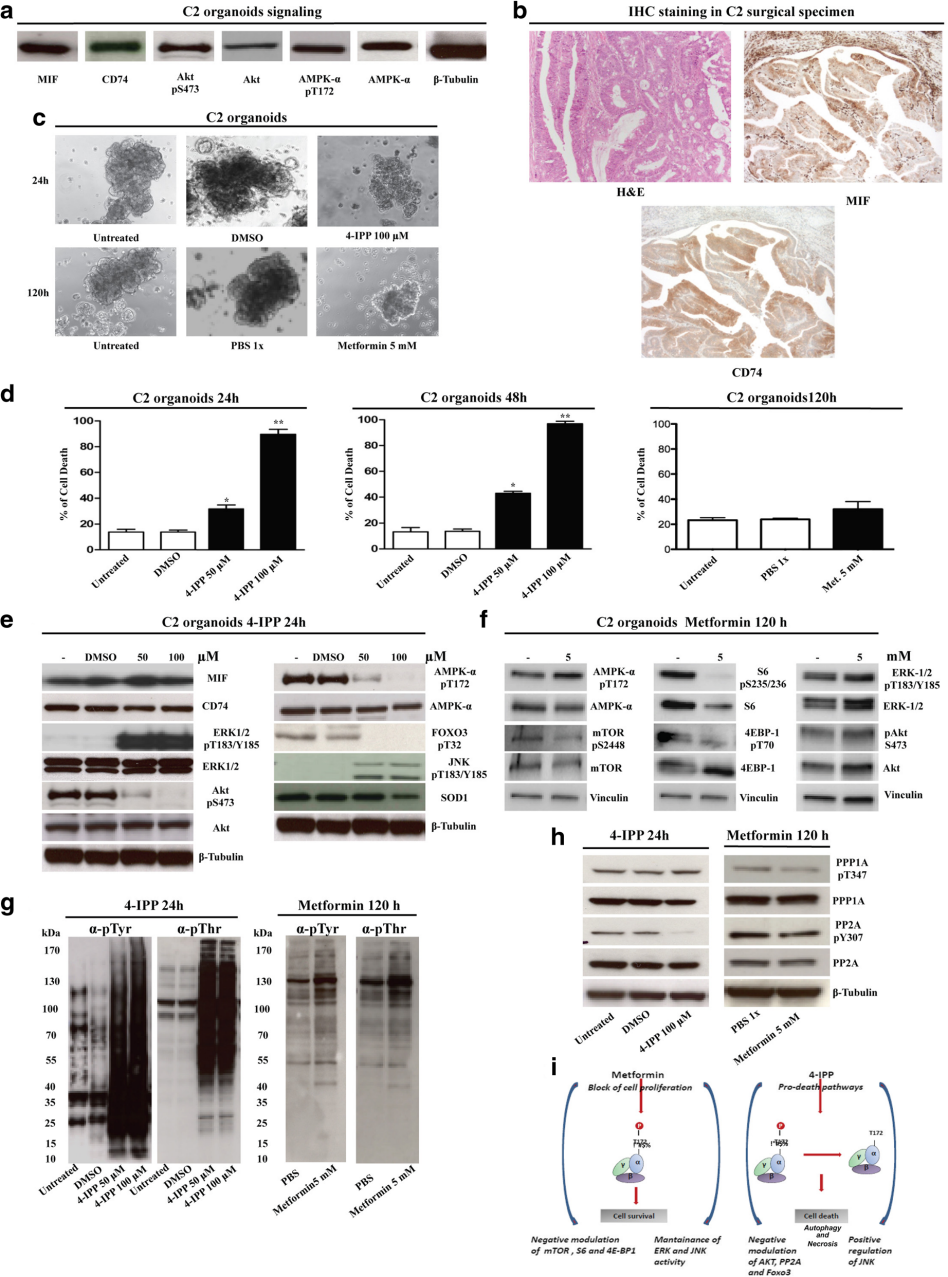
Effects of 4-IPP and metformin on C2 organoids. **a** MIF/CD74 signaling in C2 organoids. Lysates from C2 organoids expressed MIF, CD74 and presented activated AKT and AMPK. **b** Hematoxylin eosin and immunohistochemical staining of C2 tumour sample with anti-MIF and anti-CD74 antibodies. Magnifications at 10X. **c** Micrographs of C2 organoids before and after drug treatments. 4-IPP and metformin treatments induced a marked morphological change in C2 organoids, resulting in a loss of their original spheroid organization. Magnifications: 10X. Scale bars = 50 μm. **d** C2 organoids were treated with 5 mM metformin for 120 h or 50 and 100 μM 4-IPP for 24 and 48 h. The percentage of cell death was determined by trypan blue exclusion assay. Data are expressed as the mean ± SD. *P < 0.01 compared with untreated control and 50 μM 4-IPP. **P < 0.01 compared with untreated control and 100 μM 4-IPP. We have not observed significant increase in the percentage of cell death after metformin treatments compared with untreated controls. All the experiments were replicated at least three times. **e** Immunoblots of the principal proteins involved in CD74/MIF pathway. C2 organoids were untreated or treated with 50 and 100 μM 4-IPP for 24 h; lysates were resolved by 4–12% SDS–PAGE and immunoblotted. **f** Immunoblots of the principal proteins involved in metformin pathway. C2 organoids were untreated or treated with 5 mM metformin for 120 h; lysates were resolved by 4–12% SDS–PAGE and immunoblotted. **g** Global tyrosine and threonine phosphorylation changes in C2 organoids after 4-IPP and metformin treatments. C2 organoids were untreated or treated with 50 and 100 μM 4-IPP for 24 h and with 5 mM metformin for 120 h; lysates were resolved by 4–12% SDS–PAGE and immunoblotted. **h** Immunoblots showing the expression and activity of the PP2A and PP1 proteins before and after 4-IPP or metformin treatments. Blots are representative of the results from multiple (at least two) experiments. **i** Observed consequences of the 4-IPP or metformin treatments on C2 organoids

MIF and CD74 were expressed in C2 organoids and in the corresponding tumour tissue (Fig. 4a and b). We then characterized key molecules involved in the downstream-signalling cascade of the MIF/CD74 complex. As expected C2 organoids exhibited AKT- and AMPK-specific phosphorylation (respectively at positions S473 and T172, Fig. 4a) and suppression of JNK activity due to MIF mediated inhibition of JAB1 (data not shown).

Since MIF/CD74 axis through AMPK activation accelerates glucose uptake, which enhances mitochondrial functions [23], we assessed whether the MIF/CD74 axis supports and maintains high levels of metabolism of C2 organoids. We have recently observed an extensive alteration of some crucial oncogenic signals and a decrease in Krebs cycle activity through the specific inhibition of MIF activity using 4-IPP in papillary and anaplastic thyroids carcinoma models [24]. In the work we demonstrated by MALDI-TOF analysis that 4-IPP can effectively recognize MIF as its target.

We expect that targeting MIF/CD74 axis in CRC-pc models could give similar results inducing a strong impairment in many crucial metabolic pathways and leading to a dramatic change of the homeostasis of colonic carcinomatosis. Conversely, we examined the effects of AMPK stimulation by using metformin, an AMPK activator that disrupts mitochondrial functions through inactivating mitochondrial respiratory chain complex I [25].

### Cellular and signalling effects of 4-IPP and Metformin on C2 organoids

We evaluated the 4-IPP effects by targeting C2 organoids with various concentrations of inhibitor (10, 25, 50, 100 μM). Twenty-four hours after treatment with 4-IPP, C2 organoids appeared disaggregated (Fig. 4c left) and died within 48 h. The disaggregation of organoids was also observed after treatment with metformin that, on the other hand, induced only a slight increase of cell death after 120 h (Trypan blue assay, Fig. 4d).

Treatment with 50 and 100 μM 4-IPP activated ERK phosphorylation. In contrast, AKT and AMPK phosphorylation was abrogated (Fig. 4e). The decreased phosphorylation of the AKT-specific phosphorylation site on FOXO3 further demonstrated the suppression of AKT activity and possibly supported the impairment of C2 viability. Shorter 4-IPP treatments (3, 6 and 12 h) exhibited similar results (Additional file 2: Figure S3). Treatment of C2 organoids with metformin inhibited phosphorylation of mTOR, ribosome protein S6 and 4E-BP1 but did not inhibit ERK and AKT (Fig. 4f). Treatments with 4-IPP increased the activity of the stress-activated protein kinase, JNK, in a dose-dependent manner, explainable by the activation of a complete stress-related response (Fig. 4e). In parallel, a dramatic change in the anti-phosphotyrosine/phosphothreonine profile was observed probably due to inhibited phosphatase activities (Fig. 4g, left panel). Indeed, PP2A phosphorylation was switched off after 4-IPP treatment (Fig. 4h, left panel). Conversely, metformin treatment did not significantly modify phosphotyrosine/phosphothreonine profiles and phosphorylation of PP2A and PPP1A (Fig. 4g, and h, right panels).

These results suggested that inhibition of AKT and AMPK-related pathways has more pronounced effects in terms of cell survival and stress-related response rather than the implementation of AMPK/mTOR/S6/4EBP1 axis (Fig. 4i).

### Metabolic changes after 4-IPP and metformin treatments

**Fig. 5:**
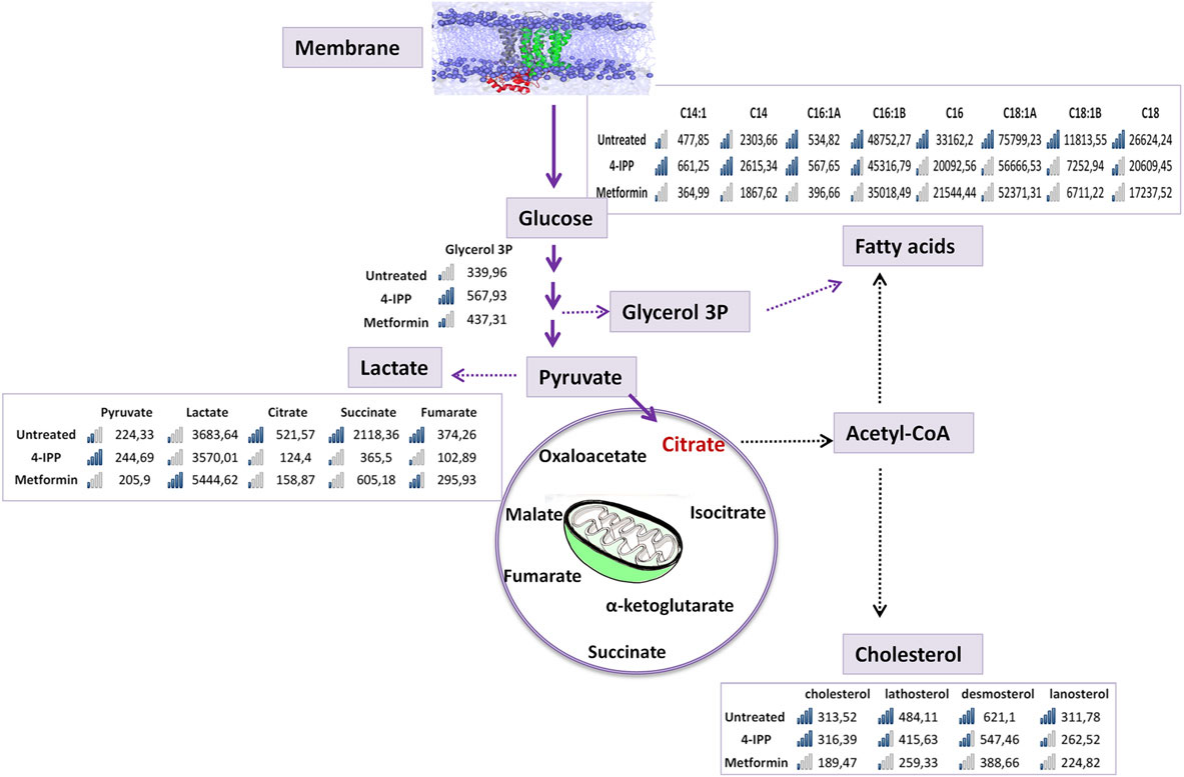
Metabolic changes after 4-IPP or metformin treatments. The figure shows the bargraphs relative of the metabolites analysed by mass spectrometry after C2 treatments with 4-IPP at 100 μM for 24 h and metformin at 5 mM for 120 h. Values are expressed as ng/mg proteins and are normalized to the value of total proteins present in each sample. Each value represents the mean of five independent replicates. Metabolites values are also plotted; blue bars mark the amount with respect to the highest values. Metabolic organic acids intermediates of the TCA cycle (citrate, succinate and fumarate) were reduced both in 4-IPP and metformin treated cells. In the case of metformin treated cells, there was a significant increase in lactate production, suggestive of an inefficient pyruvate-dehidrogenase activity due to impaired TCA and OXPHOS. Citrate released by mitochondria is converted into Acetyl-CoA, NADPH and ATP for lipid (cholesterol and fatty acids) synthesis. Cholesterol and precursor sterols (markers of cholesterol synthesis) were reduced in both 4-IPP and metformin treated cells. Very long chain fatty acids (>C16) were reduced by both treatments

To investigate the metabolic effects of the two drugs, we analysed the cellular amount of some metabolites involved in glycolysis, tricarboxylic acid (TCA) cycle, fatty acids (FAs) and cholesterol synthesis in lysates from C2 organoids after 4-IPP and metformin treatments by isotope-dilution gas chromatography-mass spectrometry (GC-MS) analysis [15]. After 4-IPP treatment, glycerol-3-phosphate and pyruvate were elevated and citrate, fumarate, malate and succinate were reduced (Fig. 5 and Additional file 1: Table S3). Conversely, after metformin treatment, lactic acid and glycerol-3-phosphate were elevated and pyruvate, succinate, fumarate, malate and citrate were reduced. 4-IPP treated C2 organoids showed a moderate reduction in fatty acids and cholesterol synthesis, while exposure to metformin resulted in reduced fatty acid levels and cholesterol synthesis that were more pronounced than that observed in cells treated with 4-IPP (Fig. 5 and Additional file 1: Table S3). Metformin treatment reduced cholesterol synthesis (measured as the cellular amount of cholesterol precursor lathosterol, lanosterol and desmosterol), as well as the amount of cholesterol and membrane fatty acids which are part of phospholipids, structural molecule of the lipid bilayer. It is likely that the alterations to the cholesterol and fatty acid metabolism may be both the consequence as well as a mechanism to induce the mitochondrial dysfunction in tumour cells exposed to metformin. Moreover the increase of pyruvate followed by the strong impairment in the production of the principal metabolites involved in the TCA cycle in C2 organoids after 4-IPP treatment seems to be related to a mitochondria disruption provoked by the knock down of MIF activity.

### Mitochondria impairment after 4-IPP and metformin treatments in C2 organoids

**Fig. 6:**
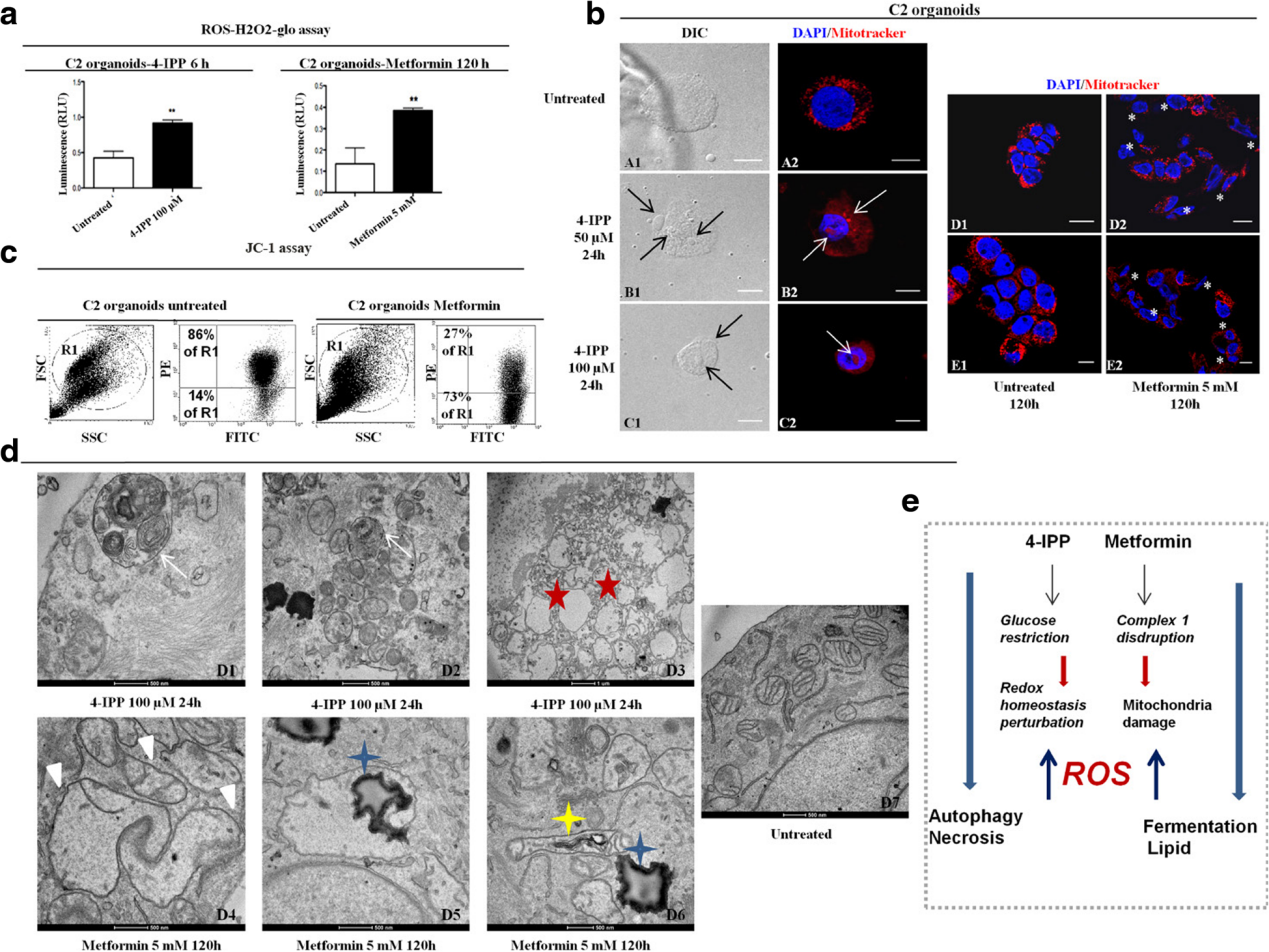
Mitochondria impairment after 4-IPP and metformin treatments in C2 organoids. **a** Reactive oxygen species detection with ROS-H2O2-Glo assay kit. C2 cells were treated with metformin 5 mM, 4-IPP 100 μM or left untreated for 120 and 24 h respectively. Extracellular H2O2 formation was detected and quantified using the ROS-H2O2-Glo assay. Luminescence intensity was quantified using a microplate reader with a 500 ms integration time, reported as relative light units and normalised to 0 cells/well treatment conditions. Standard deviation was calculated for a set of triplicate values. **b** Confocal microscopy images of mitochondria using Mitotracker® Deep Red FM in C2 cells untreated (**A2**; **D1**; **E1**), treated with 50 μM 4-IPP for 24 h (**B2**), 100 μM 4-IPP for 24 h (**C2**), or 5 mM metformin for 120 h (**D2**; **E2**). Arrows indicate aggregated mitochondria. 4-IPP treatment leads to mitochondria impairment in C2 cells. Metformin treatment reduces signal intensity of Mitotracker (asterisk) suggesting a depolarization of mitochondrial membrane potential. The figure shows data from a representative experiment. DIC: differential interference contrast. Magnifications: 10X. Scale bars = 50 μm. All the experiments were replicated at least three times. **C** Flow cytometry analysis of the mitochondrial membrane potential using JC-1 assay on C2 organoids treated with metformin for 120 h or left untreated. Staining JC-1 revealed that metformin treatment caused a significant increase in the amount of FITC aggregates, a pattern that is characteristic for disruption of mitochondrial membrane potential. **d** The ultrastructure of the C2 cells after 4-IPP (**D1-3**) or metformin treatment (**D4-6**) and left untreated (**D7**). The white arrows show autophagic structures and red stars indicate apoptotic bodies present in 4-IPP treated cells. White triangles show irregular mitochondria, blue stars lipid droplets and the yellow star a multilamellar structure present in metformin treated cells. Additionally, no signs of autophagy, apoptosis or irregular mitochondria were observed in untreated cells (**D7**). **e** Functional consequences of 4-IPP or metformin treatments on C2 organoids

Because mitochondria are known to produce significant amounts of ROS that may generate oxidative stress contributing to mitochondrial damage, affecting signal transduction pathways, cellular functions and viability, we studied the effects of the two compounds on ROS production (Fig. 6a). ROS-H_2_O_2_-Glo assay showed an increase of ROS production in C2 organoids after metformin and 4-IPP treatments (2–2.5 times). This result suggests that C2 organoids undergo genotoxic stress due to rising levels of ROS.

A mitochondrial-targeted probe which accumulates in mitochondria, depending on their membrane potential, was used to morphologically explore the mitochondrial network in C2 organoids under 4-IPP and metformin inhibition. Briefly, we monitored the mitochondrial membrane potential using JC1 probe, a cationic carbocyanine fluorescent dye that exhibits a potential-dependent accumulation in mitochondria: a fluorescent emission shift from green (~525 nm) to red (~590 nm) indicates increase of the membrane potential, while a decrease in the red/green fluorescence intensity ratio highlights mitochondrial depolarization. Our analyses showed that mitochondria were distributed broadly in the cytoplasm and characterized by a perinuclear punctuated network in untreated cells (Fig. 6b). Cells treated with 4-IPP showed mitochondria with an altered morphology indicated by changes in immunofluorescence staining patterns. Mitochondria appeared much less tubular and a diffuse cytosolic pattern of fluorescence was observed. Of note, treated cells also showed signs of nuclear destruction (Fig. 6b, left). Metformin treatment decreased the fluorescence of the probe and several cells displayed a negative fluorescence signal (Fig. 6b, right), indicating a clear loss of mitochondrial membrane potential, which is an important indicator of functional mitochondria. The results highlighted a mitochondrial depolarization after metformin treatment (Fig. 6c).

Using electron microscopy to examine the morphology of mitochondria in C2 organoids treated with 4-IPP, we observed signs of necrosis, including strong condensation of chromatin along a nuclear envelope. There, mitochondria were smaller than in non-treated cells with significant impairment of their structure. The number of lipid droplets in cytosol was smaller than in cells treated with metformin (Fig. 6D1-D3). In organoids treated with metformin, we found many large mitochondria with irregular contours and a modest number of cristae. These mitochondria contained multi-lamellar structures inside, and were aligned with residual lipid droplets accumulated inside the cytosol (Fig. 6D4-D6).

These results suggested that ROS production likely due to the loss/reduction of mitochondrial activity affected viability of C2 organoids (Fig. 6e). Specifically, 4-IPP treated C2 organoids showed autophagy structures (Fig. 6d) that are associated with necrosis and cell death.

### 4-IPP and metformin effects are recapitulated also in the C1 organoids

**Fig. 7:**
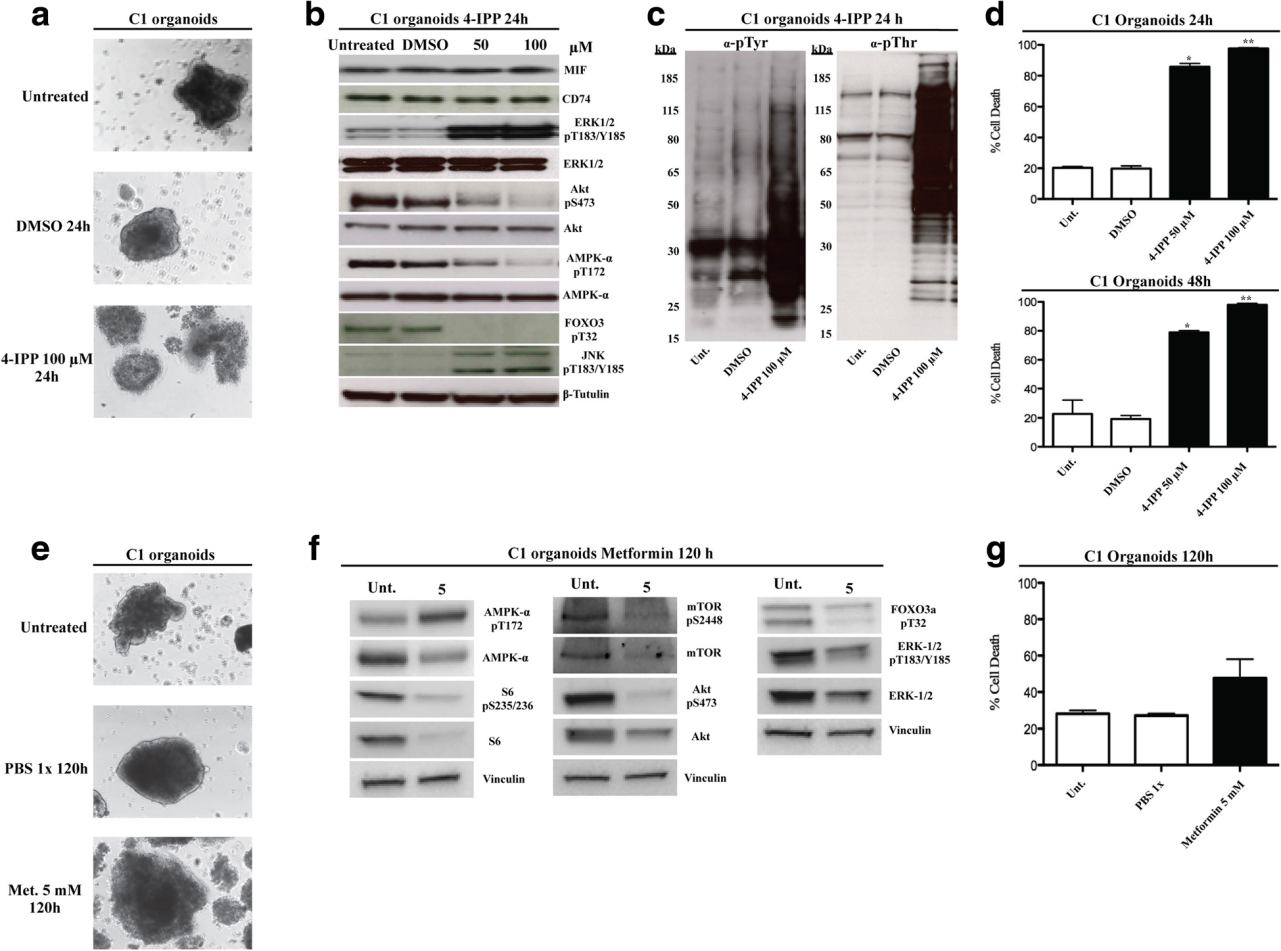
4-IPP and metformin effects are recapitulated using C1 organoids. **a** Micrographs of C1 organoids before and after 4-IPP treatments. 4-IPP treatment induced loss of the original spheroid organization. Magnifications: 10X. Scale bars = 50 μm. **b** Immunoblots of the principal proteins involved in CD74/MIF signaling pathway. C1 organoids were untreated or treated with 50 and 100 μM 4-IPP for 24 h; lysates were resolved by 4–12% SDS–PAGE and immunoblotted. **c** Immunoblots showing the expression and activity of the α-pTyr and α-pThr proteins before and after 4-IPP treatment. C1 organoids were untreated or treated with 50 and 100 μM 4-IPP for 24 h; lysates were resolved by 4–12% SDS–PAGE and immunoblotted. **d** C1 organoids were treated with 50 and 100 μM 4-IPP for 24 and 48 h. The percentage of cell death was determined by trypan blue exclusion assay. Data are expressed as the mean ± SD. *P < 0.01 compared with untreated control and 50 μM 4-IPP. **P < 0.01 compared with untreated control and 100 μM 4-IPP. All the experiments were replicated at least three times. **e** Micrographs of C1 organoids before and after metformin treatments. Metformin treatment induced a moderate loss of the original spheroids organization. Magnifications: 10X. Scale bars = 50 μm. **f** Immunoblots of the principal proteins involved in the metformin pathway. C1 organoids were treated with 5 mM metformin for 120 h; lysates were resolved by 4–12% SDS–PAGE and immunoblotted. Images are representative of the results from at least two experiments. **g** C1 organoids were treated with 5 mM metformin for 120 h. The percentage of cell death was determined by trypan blue exclusion assay. Data are expressed as the mean ± SD. We have not observed significant increase in the percentage of cell death after metformin treatments compared with untreated controls. All the experiments were replicated at least three times

To further evaluate the relevance of the results obtained through 4-IPP and metformin, we assessed whether 4-IPP and metformin could inhibit the MIF/CD74 signalling axis and AMPK, respectively, by determining their effects on the expression of targets also in C1 organoids. Treatments impaired the principal morphological features of C1 organoids, leading to loss of the spheroids organization (Fig. 7a and d). Western blot analysis confirmed that 4-IPP at 50 and 100 μM increased ERK-1/2 phosphorylation and down-regulated AKT and AMPK activity (Fig. 7b). Moreover in C1 organoids, 5 mM metformin inhibited the mTOR pathway, and downregulated AKT and ERK activity (Fig. 7e). As shown in Fig. 7c, the anti-phosphotyrosine and anti-phosphothreonine profiles of the 4-IPP-treated cells presented a significant increase in phosphorylated proteins profiles compared with control groups. In parallel, we observed the change of PP2A phosphorylation status indicating failed phosphatase activity (data not shown). Trypan blue assay also displayed that 4-IPP treatments at 50 and 100 μM, strongly impaired C1 organoids viability after twenty-four and forty-eight hours. On the other hand, after one-hundred and twenty hours, 5 mM metformin slightly affected C1 viability (Fig. 7d). Taken together, these results confirm the validity and reproducibility of treatments with 4-IPP and metformin in colon-cancer carcinomatosis-derived organoids.

**Fig. 8:**
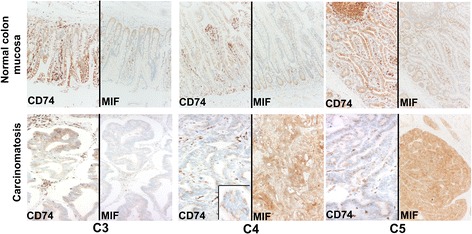
CD74 and MIF IHC staining in three additional CRC-pc samples. In the upper part of the Figure, the normal colon mucosa from the three patients is shown. In all the cases, CD74 is highly expressed in epithelial cells and in the surrounding normal cellular microenvironment. Conversely, MIF is poorly expressed. MIF staining is mainly restricted to epithelial cells. In the lower part of the figure, the corresponding peritoneal carcinomatoses (C3, C4 and C5) are shown. Immunohistochemical staining for CD74 is heterogeneous with a granular, cytoplasmic and membranous pattern in C3, while focal, granular and restricted to the inner luminal aspect in C4 (inset) and C5. MIF immunostaining is homogeneous and very low expressed in C3 compared to C4 and C5. MIF staining is mainly cytoplasmic. Images were taken using a Nikon Eclipse 80i microscope (Nikon, Tokyo, Japan) and Nikon digital sight DS-Fi1 camera equipped with control unit-DS-L2 (Nikon). Images were assembled using Adobe Photoshop 6 (Adobe Systems, San Jose, CA, USA). Original magnification at 20X

Immunohistochemical expression of CD74 and MIF was studied in three additional peritoneal CRC-pcs (C3, C4 and C5), along with their corresponding normal colon samples (Fig. 8). In normal colon samples, CD74 was widely and strongly expressed by both normal epithelial cells and the surrounding reactive cell microenvironment. The lymphocytes were strongly CD74 positive. The expression of CD74 in neoplastic tumour cells was heterogeneous, ranging from strong cytoplasmic and membranous (samples C4 and C5) to weak granular cytoplasmic (sample C3). MIF was poorly expressed by normal epithelial cells. Conversely, all tumour cells exhibited cytoplasmic and membranous staining which ranged from weak to moderate and strong.

## Discussion

We explored the opportunity of using data from the secretome analysis of patient-derived cancer organoids established from two peritoneal carcinomatosis (C1 and C2), to identify possible targets for therapy. The analysed organoids presented self-renewal capacity, the ability to form the typical 3D structure, and a significant positivity for stem biomarkers, as shown by the number of highly-dividing LGR5+ CSCs, especially in C2 organoids. LGR5+ cells are believed to be fast-cycling and resistant to irradiation and chemotherapy and have been shown to initiate intestinal organoid growth in a 3D culture system when isolated from the mouse small intestine [8, 26]. This high proliferation suggests that LGR5+ cells are adapted to facilitate the uptake and incorporation of nutrients into the biomass (nucleotide, amino acids and lipids) needed to generate new cells [27]. Remarkably, this adaptation is not merely due to in vitro growth conditions but was a characteristic already present in the original tumours, both presenting a high number of LGR5+ cells and high mitotic index.

Accordingly, GO Analysis of the secretome profile of the fastest growing C2 organoids revealed prominent expression of metabolic pathways related to oxidative homeostasis, glucose metabolism, and the major glucose catabolic pathway (pentose phosphate pathway) that links glucose metabolism to biosynthesis of the nucleotide precursor ribose, coordinating glucose flux and supporting the cellular biogenesis of macromolecules and energy production [28].

The simultaneous positivity of the two organoids for MIF suggests the rationale for inhibition of MIF/CD74 signalling axis, which is known to support AMPK activation, glycolysis, oxidative phosphorylation, but also to compensate for the effects of high glucose oxidation [29]. AMPK activation enhances both the transcription and translocation of Glut4 with a consequent increase in glucose uptake, which in turn activates the PI3K/AKT pathway [30]. Activated AMPK is also responsible for initiating alternative energy-generating processes such as fatty acid oxidation [31]. The activation of AMPK and the subsequent glycolytic switch observed in mitotic cells supports the possibility of a dependence on energy pathways to survive in mitosis [32]. AMPK senses ATP loss and enhances glycolysis to protect from death. Previous research has shown that MIF acts through the AMPK signalling pathway to induce cellular resistance to glucose deprivation, ischemia, hypoxia, oxidation and stress senescence [22].

Our results support the concept that CSCs, through the expression of MIF and CD74, become protected by energy-default processes which force cancer cells with defective mitochondria to generate energy through an AMPK-regulated glycolysis in the cytoplasm [33]. Thus, the inhibition of AMPK signalling through the suppression of MIF/CD74 signalling could be a rational strategy that affects the special energetic requirements of C1 and C2 organoids.

Indeed, knocking down MIF/CD74 signalling through 4-IPP decreased SOD1 and resulted in changes in mitochondrial morphology and mass. An increase of ROS produced a JNK mediated stress response (Fig. 4). The oxidative shift in the redox conditions reduced phosphatases activity, blocking signal propagation in the cell through a number of signalling pathways involving protein kinases [34].

In both organoids, the inhibition of AMPK-mediated signalling is associated with the inactivation of both AKT and FOXO3. Of note, FOXO3 can induce a number of genes that protect against ROS suggesting that it plays a role in keeping cellular ROS low. JNK activation resulted after treatment, possibly modulating cellular response to ROS independent of changes in the intracellular AMP level [7] and likely inducing a defective autophagic process followed by rapid cell death.

ROS increase is also the cause of the observed inactivation of PP2A phosphatase and of the relevant consequences in terms of signal transduction response. Inhibition of ERK1/2 dephosphorylation and the general inhibition of protein dephosphorylation observed after treatment support this interpretation of the results.

However, redox regulation of phosphatases is poorly understood. Protein phosphatases are inhibited by ROS, which target specific redox sensitive amino acids, but PP2A catalytic activity leading to the activation of cellular stress signalling pathways can be also modulated by hydrogen peroxide [35].

Our findings define a MIF/CD74/AMPK pathway in the colon peritoneal carcinomatosis, for the first time elucidating a signal transduction mechanism between autocrine/paracrine factors and AMPK activation. The results provide initial proof-of-concept that pharmacologic treatment blocking MIF/CD74 signalling pathways disrupts energy homeostasis in CRC-pc derived organoids, in vitro, by inducing cell death.

Our investigations were also aimed to study the effects of metformin, a drug for treating type 2 diabetes, whose use has been associated with decreased cancer risk, and that kills cancer-initiating/stem cells from a variety of cancers with AMPK activation [25]. Metformin is known to inhibit the mitochondrial respiratory chain complex I that reduces ATP production and increases the intracellular ratio of AMP/ATP, resulting in AMPK activation that switches cells from an anabolic to a catabolic state. We found that metformin reprograms organoid metabolism. This process involved both AMPK activation, which reduces mTOR activity and protein translation, and complex I inhibition, which promotes ROS production within the mitochondrial matrix. As an effect of AMPK activation, glycolysis was stimulated, producing a large amount of lactate. Furthermore, the inhibition of complex I promoted superoxide production within the mitochondrial matrix, which provoked dysfunctional mitochondrial swelling, interrupted the tricarboxylic acid cycle and led to a compensatory increase in lactate and glycolytic ATP and aberrant lipid accumulation.

C2 organoids treated with metformin presented dramatic changes in mitochondria morphology – deformation and partial loss of mitochondrial membranes and degradation of cristae. Consequently, we observed accumulation of free radicals and loss of membrane potential. These effects could be explained by the ability of metformin to induce mitochondrial depolarization and increase ROS through inhibition of complex I [36], which also leads to mitochondrial cristae degradation [37]. Loss of mitochondrial function limits the production of lipids, nucleotides and amino acids used by cells for the creation of new biomass, and could explain the inhibited cell proliferation after treatment with metformin, as suggested by the reduced number of disaggregated cells counted in treated samples during the trypan blue assay (data not shown).

In conclusion, 4-IPP and metformin have profound effects on the metabolism of organoids derived from CRC-pcs. Interestingly, C1 and C2 organoids presented different phenotypic features, but similar responses when treated with 4-IPP or metformin – activation of stress-signalling pathways and cell death with 4-IPP treatments; inhibition of mitochondria, oxidation of glucose and lipids resulting in energy restriction with metformin treatments.

We have previously shown that inhibition of MIF/CD74 signalling axis blocks thyroid cancer cells growth by inducing cell death during mitosis [24]. Other studies have demonstrated the efficacy of MIF or CD74 targeting in several cancers providing a rationale for the development of therapeutic agents and clinical trials to study the effects of inhibiting a newly discovered MIF isoform (oxMIF) on different cancers, including metastatic CRC (NCT01765790). Milatuzumab, the first anti-CD74 antibody that has entered into clinical tests, is currently being studied for the treatment of non Hodgkin lymphomas, chronic lymphocytic leukemia, and multiple myeloma [33, 38] and could be potentially tested in MIF/CD74 positive CRC-pc.

MIF inhibitors, particularly when acting on the MIF/CD74 axis, could be a very attractive strategy for cancer therapeutic intervention due to the nonphysiologic enzymatic activity of the cytokine that is evolutionary well-conserved [39]. MIF belongs to the family of tautomerase proteins and can catalyze the tautomerization of nonphysiologic substrates, such as D-dopachrome and L-dopachrome methyl ester, into their corresponding indole derivates. This peculiar feature seems to be mediated by the NH_2_-terminal Proline of MIF (Pro-1) and strategies based on targeting Pro-1 are able to abrogate the tautomerase activity [40]. Indeed, different works have shown that the development of small molecules antagonist of the MIF enzymatic active site could be an effective strategy to abrogate its functions. Winner and colleagues showed how 4-IPP serves as suicide substrate for MIF, resulting in the covalent modification of Pro-1 [41]. Exposure to 4-IPP decreased MIF immunoreactivity and similar results were also recapitulated using MIF silencing-based strategies [42]. Some recent works showed how 4-IPP interferes with several activities peculiar of MIF, such as neutrophil lung recruitment [43], the maintenance of a chemoresistant phenotype [44] and the reprogramming of tumor associated macrophages that supports malignant plasma cell survival and resistance to therapy in multiple myeloma cells [45].

Moreover, MIF inhibitors, particularly in conjunction with CD74 inhibition, could be very efficient in blocking CRC-pc progression. The systemic effects of inhibitors that interfere with energy metabolism are still only partially known, however ongoing clinical studies seem to give encouraging results.

Differently, metformin is widely used with mild or no side effects, and therefore the effects of energetic reduction induced by metformin on organoids could be combined with drugs which can act in synergy and induce cell death.

## Conclusions

Our study shows that the modulation of AMPK function, both through its activation by metformin, or its reduction caused by the removal of MIF/CD74 signalling axis, is critical for maintaining cell energy balance of CRC-pc. Metformin elicits the metabolic reprogramming by decreasing oxidative phosphorylation and increasing dependency on glycolytic metabolism. Conversely, inhibition of MIF/CD74 signalling axis elevated ROS rendering cancer cells vulnerable to oxidative stress-induced cell death. In conclusion, targeting the metabolism of cancer cells opens new lines of research that could lead to the design of new therapies for the treatment of CRC-pc.

## Additional files

Additional file 1: Table S1. Antibodies for immunohistochemistry. **Table S2**. Antibodies for Western blot. **Table S3**. Metabolic changes after 4-IPP or metformin treatments. (PDF 313 kb)
Additional file 2:
**Figure 1.** Phase contrast microscope images of C1 and C2 organoids. **Figure 2.** Ingenuity Pathway Analysis of C2 secretome. **Figure 3.** Western blot analysis of C2 organoids after 4-IPP treatments. (PDF 282 kb)

## Abbreviations

3D: Three-Dimensional
4-IPP: 4-iodo-6-phenylpyrimidine
AMP: Adenosine monophosphate
ATP: Adenosine triphosphate
C1: Colorectal cancer peritoneal carcinomatosis organoid 1
C2: Colorectal cancer peritoneal carcinomatosis organoid 2
C3: Colorectal cancer peritoneal carcinomatosis organoid 3
C4: Colorectal cancer peritoneal carcinomatosis organoid 4
C5: Colorectal cancer peritoneal carcinomatosis organoid 5
CoA: Coenzyme A
CRC: Colorectal cancer
CRC-pc: Colorectal cancer peritoneal carcinomatosis
CSCs: Cancer Stem Cells
DAB: 3, 3′-diaminobenzidine
DMSO: Dimethyl sulfoxide
Fas: Fatty acids synthesis
FDR: False discovery rate.
FFPE: Formalin Fixed Paraffin Embedded
GC-MS: Isotope-dilution gas-chromatography mass spectrometry
GO: Gene Ontology
H&E: Haematoxylin and eosin
H2O2: Hydrogen peroxide
ISH: in situ Hybridization
JC-1: 5′,6,6′-tetrachloro-1,1′,3,3′-tetraethylbenzimidazolylcarbocyanine iodide
lc-MS/MS: Nano-scale liquid-chromatography tandem mass spectrometry
LGR5: Leucine-rich repeat-containing G-protein coupled receptor 5
MIF: Macrophage Migration Inhibitory Factor
NADPH: Nicotinamide adenine dinucleotide phosphate
OsO4: Osmium tetroxide
oxMIF: Oxidized Macrophage Migration Inhibitory Factor isoform
OXPHOS: Oxidative phosphorylation
PANTHER: Protein Analysis Through Evolutionary Relationships
PD: Parkinson’s disease
pThr: Phospho-threonine
pTyr: Phospho-tyrosine
ROS: Reactive Oxygen Species
SD: Standard deviation
SDS: Sodium dodecyl sulfate
STRING: Search Tool for the Retrieval of Interacting Genes/proteins
TCA: Tricarboxylic acid cycle
