# Correction: MIF/CD74 axis is a target for novel therapies in colon carcinomatosis

**DOI:** 10.1186/s13046-026-03801-8

**Published:** 2026-08-19

**Authors:** Fabio Bozzi, Angela Mogavero, Luca Varinelli, Antonino Belfiore, Giacomo Manenti, Claudio Caccia, Chiara C. Volpi, Galina V. Beznoussenko, Massimo Milione, Valerio Leoni, Annunziata Gloghini, Alexandre A. Mironov, Ermanno Leo, Silvana Pilotti, Marco A. Pierotti, Italia Bongarzone, Manuela Gariboldi

**Affiliations:** 1 Department of Diagnostic Pathology and Laboratory Medicine, Fondazione IRCCS Istituto Nazionale dei Tumori, via G. Venezian 1, Milan, 20133 Italy; 2 Department of Experimental Oncology and Molecular Medicine, Fondazione IRCCS Istituto Nazionale dei Tumori, via G. Amadeo 42, Milan, 20133 Italy; 3 Molecular Genetics of Cancer, Fondazione Istituto FIRC di Oncologia Molecolare, via Adamello 16, Milan, 20139 Italy; 4 Proteomics Laboratory Department of Experimental Oncology and Molecular Medicine, Fondazione IRCCS Istituto Nazionale dei Tumori, via G. Amadeo 42, Milan, 20133 Italy; 5 Department of Predictive and Preventive Medicine, Fondazione IRCCS Istituto Nazionale dei Tumori, via G. Amadeo 42, Milan, 20133 Italy; 6 Laboratory of Clinical Pathology and Medical Genetics, Fondazione IRCCS ‘Carlo Besta’ Istituto Neurologico, via G. Amadeo 42, Milan, 20133 Italy; 7 Fondazione Istituto FIRC di Oncologia Molecolare, via Adamello 16, Milan, 20139 Italy; 8 Colorectal Cancer Unit-Department of Surgery, Fondazione IRCCS Istituto Nazionale dei Tumori, via G. Venezian 1, Milan, 20133 Italy

**Correction: BMC Plant Biol 36, 16 (2017)**

**https://doi.org/10.1186/s13046-016-0475-z**

Following publication of the original article [1], the authors recognized that lanes 3–4 of Fig. 4e overlap with lanes 1–2 of Fig. 7b. This overlap resulted from an unintentional mistake made while assembling, cropping, and editing the figures, which led to overlap or duplication in a few instances.

After carefully checking the original data, the authors confirmed that they mistakenly selected the images during the process of image editing and cropping. The authors provided the correct western blots of C1 organoid experiments, which reproduce the same experimental findings and confirm the robustness of the reported results. Since the error does not originate from original experiments, this correction does not affect the overall conclusion of this article.

**Incorrect Figure 7**

**Fig. 7:**
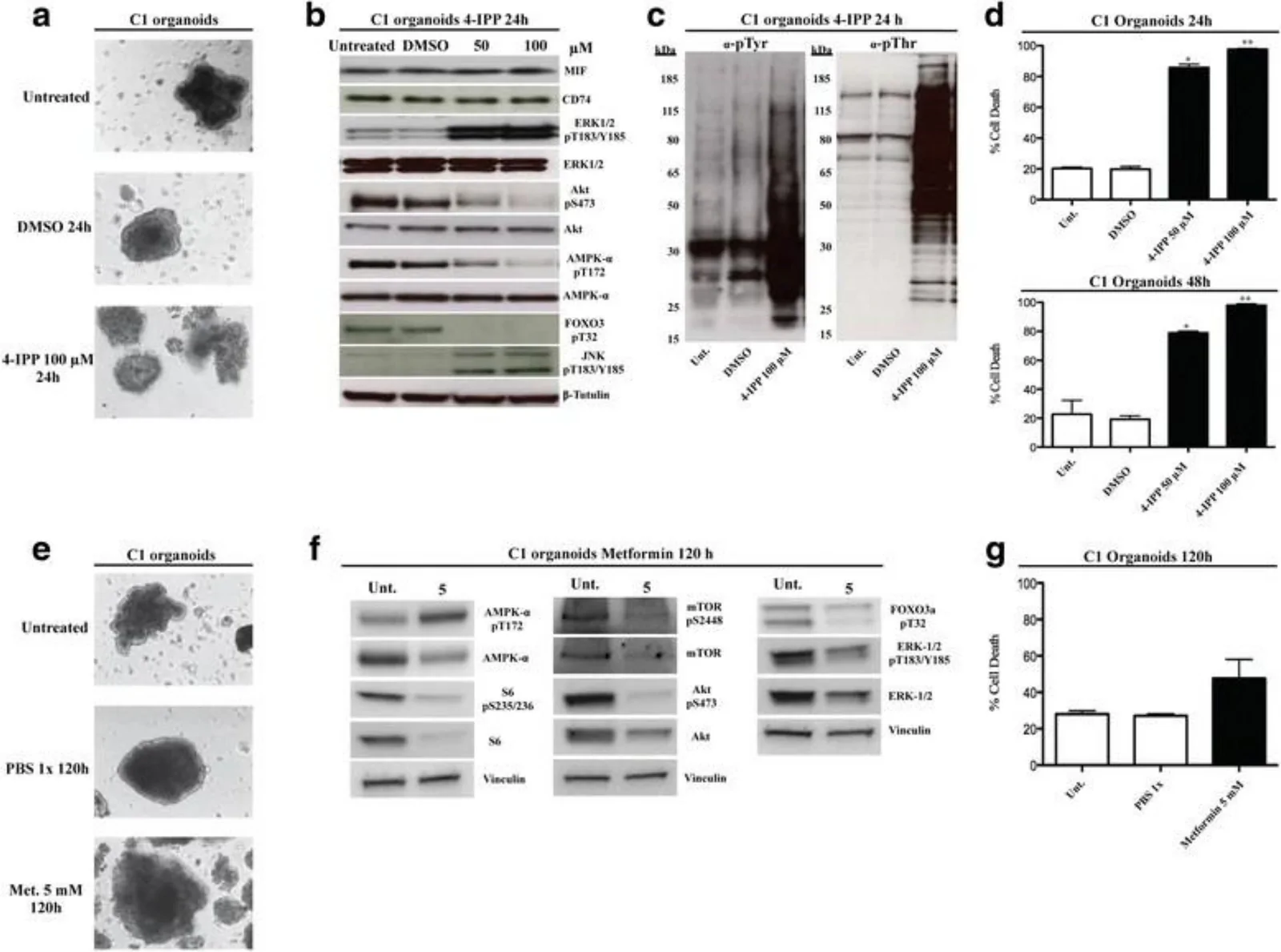
4-IPP and metformin effects are recapitulated using C1 organoids. **a** Micrographs of C1 organoids before and after 4-IPP treatments. 4-IPP treatment induced loss of the original spheroid organization. Magnifications: 10X. Scale bars = 50 μm. **b** Immunoblots of the principal proteins involved in CD74/MIF signaling pathway. C1 organoids were untreated or treated with 50 and 100 μM 4-IPP for 24 h; lysates were resolved by 4–12% SDS–PAGE and immunoblotted. **c** Immunoblots of p-ERK1/2 Thr183/Tyr185. C1 organoids were untreated or treated with 10, 25, 50 and 100 μM for 24 h. Total ERK1/2 was used as a control for ERK1/2 protein abundance. lysates were resolved by 4–12% SDS–PAGE and immunoblotted. **d** Immunoblots showing the expression and activity of the α-pTyr and α-pThr proteins before and after 4-IPP treatment. C1 organoids were untreated or treated with 50 and 100 μM 4-IPP for 24 h; lysates were resolved by 4–12% SDS–PAGE and immunoblotted. **e** C1 organoids were treated with 50 and 100 μM 4-IPP for 24 and 48 h. The percentage of cell death was determined by trypan blue exclusion assay. Data are expressed as the mean ± SD. *P < 0.01 compared with untreated control and 50 μM 4-IPP. **P < 0.01 compared with untreated control and 100 μM 4-IPP. All the experiments were replicated at least three times. **f** Micrographs of C1 organoids before and after metformin treatments. Metformin treatment induced a moderate loss of the original spheroids organization. Magnifications: 10X. Scale bars = 50 μm. **g** Immunoblots of the principal proteins involved in the metformin pathway. C1 organoids were treated with 5 mM metformin for 120 h; lysates were resolved by 4–12% SDS–PAGE and immunoblotted. Images are representative of the results from at least two experiments. **h** C1 organoids were treated with 5 mM metformin for 120 h. The percentage of cell death was determined by trypan blue exclusion assay. Data are expressed as the mean ± SD. We have not observed significant increase in the percentage of cell death after metformin treatments compared with untreated controls. All the experiments were replicated at least three times

**Correct Figure 7**

**Fig. 7:**
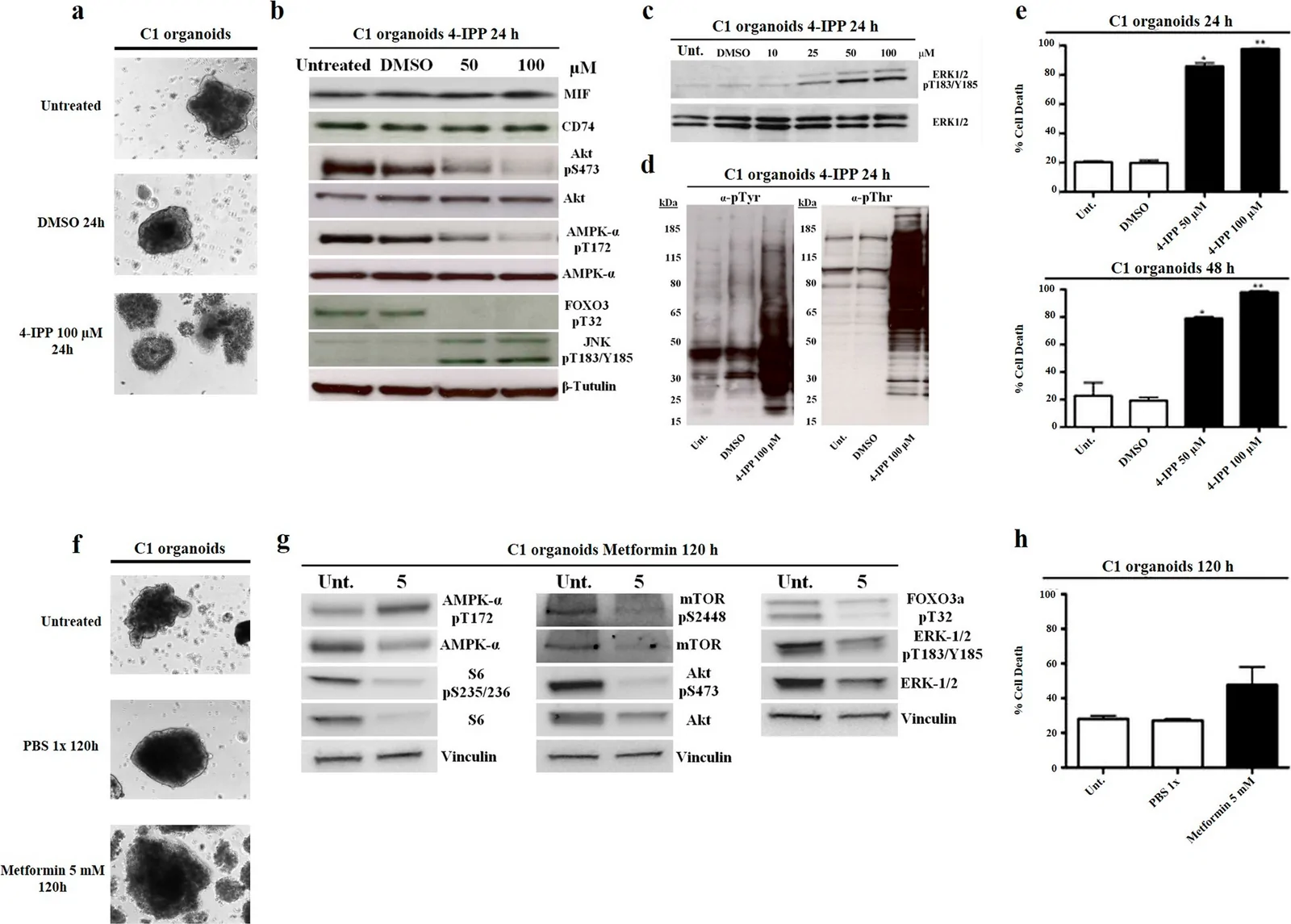
4-IPP and metformin effects are recapitulated using C1 organoids. **a** Micrographs of C1 organoids before and after 4-IPP treatments. 4-IPP treatment induced loss of the original spheroid organization. Magnifications: 10X. Scale bars = 50 μm. **b** Immunoblots of the principal proteins involved in CD74/MIF signaling pathway. C1 organoids were untreated or treated with 50 and 100 μM 4-IPP for 24 h; lysates were resolved by 4–12% SDS–PAGE and immunoblotted. **c** Immunoblots of p-ERK1/2 Thr183/Tyr185. C1 organoids were untreated or treated with 10, 25, 50 and 100 μM for 24 h. Total ERK1/2 was used as a control for ERK1/2 protein abundance. lysates were resolved by 4–12% SDS–PAGE and immunoblotted. **d** Immunoblots showing the expression and activity of the α-pTyr and α-pThr proteins before and after 4-IPP treatment. C1 organoids were untreated or treated with 50 and 100 μM 4-IPP for 24 h; lysates were resolved by 4–12% SDS–PAGE and immunoblotted. **e** C1 organoids were treated with 50 and 100 μM 4-IPP for 24 and 48 h. The percentage of cell death was determined by trypan blue exclusion assay. Data are expressed as the mean ± SD. *P < 0.01 compared with untreated control and 50 μM 4-IPP. **P < 0.01 compared with untreated control and 100 μM 4-IPP. All the experiments were replicated at least three times. **f** Micrographs of C1 organoids before and after metformin treatments. Metformin treatment induced a moderate loss of the original spheroids organization. Magnifications: 10X. Scale bars = 50 μm. **g** Immunoblots of the principal proteins involved in the metformin pathway. C1 organoids were treated with 5 mM metformin for 120 h; lysates were resolved by 4–12% SDS–PAGE and immunoblotted. Images are representative of the results from at least two experiments. **h** C1 organoids were treated with 5 mM metformin for 120 h. The percentage of cell death was determined by trypan blue exclusion assay. Data are expressed as the mean ± SD. We have not observed significant increase in the percentage of cell death after metformin treatments compared with untreated controls. All the experiments were replicated at least three times

Moreover, corrections in the body particularly under Results section, heading 4-IPP and metformin effects are recapitulated also in the C1 organoids were also corrected. The corrections are highlighted below in** bold.**

To further evaluate the relevance of the results obtained through 4-IPP and metformin, we assessed whether 4-IPP and metformin could inhibit the MIF/CD74 signalling axis and AMPK, respectively, by determining their effects on the expression of targets also in C1 organoids. Treatments impaired the principal morphological features of C1 organoids, leading to loss of the spheroids organization (Fig. 7a and **f**). Western blot analysis confirmed that 4-IPP at 50 and 100 μM **down-regulated AKT and AMPK activity and increased ERK-1/2 phosphorylation (Fig. 7b and c).** Moreover, in C1 organoids, 5 mM metformin inhibited the mTOR pathway, and downregulated AKT and ERK activity (Fig. 7**g**). As shown in Fig. 7**d**, the anti-phosphotyrosine and anti-phosphothreonine profiles of the 4-IPP-treated cells presented a significant increase in phosphorylated proteins profiles compared with control groups. In parallel, we observed the change of PP2A phosphorylation status indicating failed phosphatase activity (data not shown). Trypan blue assay also displayed that 4-IPP treatments at 50 and 100 μM, strongly impaired C1 organoids viability after twenty-four and forty-eight hours. On the other hand, after one-hundred and twenty hours, 5 mM metformin slightly affected C1 viability (Fig. 7**e and h**). Taken together, these results confirm the validity and reproducibility of treatments with 4-IPP and metformin in colon-cancer carcinomatosis-derived organoids.

## References

[1] Bozzi F, Mogavero A, Varinelli L, et al. MIF/CD74 axis is a target for novel therapies in colon carcinomatosis. J Exp Clin Cancer Res. 2017;36(1):16. 10.1186/s13046-016-0475-z. Published 2017 Jan 23. 28114961 10.1186/s13046-016-0475-z PMC5260021

