# Supplementary material for: MIF/CD74 axis is a target for novel therapies in colon carcinomatosis
Source: J Exp Clin Cancer Res. 2017 Jan 23;36:16. doi: 10.1186/s13046-016-0475-z (PMC5260021; doi:10.1186/s13046-016-0475-z)
Supplement: Additional file 1: Table S1. — Antibodies for immunohistochemistry. Table S2. Antibodies for Western blot. Table S3. Metabolic changes after 4-IPP or metformin treatments. (PDF 313 kb) [file 13046_2016_475_MOESM1_ESM.pdf]

Supplementary Table 1: Antibodies for immunohistochemistry

Claudin	Thermo Fisher Scientific, Waltham, MA, USA
CKA1-CKA3	Agilent technologies, Dako Denmark A/S, Glostrup, Denmark
CK19	Sigma-Aldrich, St Louis, USA
CK7	Agilent technologies, Dako Denmark A/S, Glostrup, Denmark
CK20	Agilent technologies, Dako Denmark A/S, Glostrup, Denmark
CDX2	Agilent technologies, Dako Denmark A/S, Glostrup, Denmark
PAX8	Proteintech Group, Inc. Rosemont, IL, USA
CD74	Abcam, Inc, Cambridge UK
MIF	Abcam, Inc, Cambridge UK
Mib1	Agilent technologies, Dako Denmark A/S, Glostrup, Denmark

Supplementary Table 2: Antibodies for Western blot

CD74	Abcam, Inc, Cambridge UK
MIF	Abcam, Inc, Cambridge UK
Phospho-JNK (Thr183/Y182)	Cell Signaling Technology, Inc., Boston, MA, USA
AKT	Cell Signaling Technology, Inc., Boston, MA, USA
Phospho-AKT (Ser 473)	Cell Signaling Technology, Inc., Boston, MA, USA
ERK 1/2	Sigma-Aldrich, St Louis, USA
Phospho-ERK ½ (Thr183/Tyr185)	Sigma-Aldrich, St Louis, USA
AMPKα	Cell Signaling Technology, Inc., Boston, MA, USA
Phospho-AMPKα (Thr172)	Cell Signaling Technology, Inc., Boston, MA, USA
mTOR	Cell Signaling Technology, Inc., Boston, MA, USA
Phospho-mTOR (Ser2448)	Cell Signaling Technology, Inc., Boston, MA, USA
S6	Cell Signaling Technology, Inc., Boston, MA, USA
Phospho-S6 (Ser240/244)	Cell Signaling Technology, Inc., Boston, MA, USA
4E-BP1	Cell Signaling Technology, Inc., Boston, MA, USA
Phospho-4E-BP1 (Thr70)	Cell Signaling Technology, Inc., Boston, MA, USA
Phospho-FOXO3a (Thr32)	Cell Signaling Technology, Inc., Boston, MA, USA
SOD1	Sigma-Aldrich, St Louis, USA
HGMB1	Sigma-Aldrich, St Louis, USA
PPP1A	Abcam, Inc, Cambridge UK

Phospho-PPP1A (Thr347)	Abcam, Inc, Cambridge UK
PP2A	Abcam, Inc, Cambridge UK
Phospho-PP2A (Tyr307)	Abcam, Inc, Cambridge UK
α–Phospho-Tyrosine	Cell Signaling Technology, Inc., Boston, MA, USA
α–Phospho-Thronine	Cell Signaling Technology, Inc., Boston, MA, USA
P53	Cell Signaling Technology, Inc., Boston, MA, USA
P21	Cell Signaling Technology, Inc., Boston, MA, USA
P27	Cell Signaling Technology, Inc., Boston, MA, USA
Cyclin D1	Cell Signaling Technology, Inc., Boston, MA, USA
PARP cleaved	Cell Signaling Technology, Inc., Boston, MA, USA
LC3B	Cell Signaling Technology, Inc., Boston, MA, USA
BAD	Cell Signaling Technology, Inc., Boston, MA, USA
Phospho-BAD (Ser112)	Cell Signaling Technology, Inc., Boston, MA, USA
Bcl-2	Cell Signaling Technology, Inc., Boston, MA, USA
β–Actin	Sigma-Aldrich, St Louis, USA
β–Tubulin	Sigma-Aldrich, St Louis, USA
Vinculin	Sigma-Aldrich, St Louis, USA
Horseradish peroxidase (HRP)-conjugated anti-mouse IgG	Bio-Rad (Missisauga, Ontario, Canada)
HRP-conjugated anti-rabbit IgG	Bio-Rad (Missisauga, Ontario, Canada)

Suuplementary Table 3, page 1 **Metabolic changes after 4-IPP or metformin treatments**

ID	file	Cell line	Type	Characteristics	[ ] µg/µl	mgprot/vol
A1	carcinomatosis1	C2-Organoids	Lysate (RIPA buffer)	Untreated (DMSO) 24h	10,11	2,022
A2	carcinomatosis2	C2-Organoids	Lysate (RIPA buffer)	Untreated (DMSO) 24h	9,48	1,896
A3	carcinomatosis3	C2-Organoids	Lysate (RIPA buffer)	Untreated (DMSO) 24h	6,3	1,26
A4	carcinomatosis4	C2-Organoids	Lysate (RIPA buffer)	Untreated (DMSO) 24h	9,58	1,916
A5	carcinomatosis5	C2-Organoids	Lysate (RIPA buffer)	Untreated (DMSO) 24h	5	1
B1	carcinomatosis6	C2-Organoids	Lysate (RIPA buffer)	Metformin 5 mM 24h	5,57	1,114
B2	carcinomatosis7	C2-Organoids	Lysate (RIPA buffer)	Metformin 5 mM 24h	5,8	1,16
B3	carcinomatosis8	C2-Organoids	Lysate (RIPA buffer)	Metformin 5 mM 24h	6,75	1,35
B4	carcinomatosis9	C2-Organoids	Lysate (RIPA buffer)	Metformin 5 mM 24h	6,32	1,264
B5	carcinomatosis10	C2-Organoids	Lysate (RIPA buffer)	Metformin 5 mM 24h	5,95	1,19
C1	carcinomatosis11	C2-Organoids	Lysate (RIPA buffer)	4-IPP (α-MIF) 100 µM 24h	2,53	0,506
C2	carcinomatosis12	C2-Organoids	Lysate (RIPA buffer)	4-IPP (α-MIF) 100 µM 24h	3,95	0,79
C3	carcinomatosis13	C2-Organoids	Lysate (RIPA buffer)	4-IPP (α-MIF) 100 µM 24h	2,42	0,484
C4	carcinomatosis14	C2-Organoids	Lysate (RIPA buffer)	4-IPP (α-MIF) 100 µM 24h	2,17	0,434
C5	carcinomatosis15	C2-Organoids	Lysate (RIPA buffer)	4-IPP (α-MIF) 100 µM 24h	3,17	0,634

Characteristics	cholesterol	lathosterol	desmosterol	lanosterol	7aOHC	7bOHC
Untreated (DMSO) 24h	310,90	492,87	666,86	310,01	1,35	2,12
Untreated (DMSO) 24h	317,72	537,28	624,23	307,19	1,93	2,07
Untreated (DMSO) 24h	311,69	495,44	567,93	316,35	1,42	2,02
Untreated (DMSO) 24h	306,34	428,57	630,34	315,04	1,52	2,14
Untreated (DMSO) 24h	320,93	466,37	616,14	310,29	1,44	2,00
Metformin 5 mM 24h	183,46	266,03	351,35	220,69	1,47	2,17
Metformin 5 mM 24h	192,48	273,24	369,88	233,12	1,70	1,92
Metformin 5 mM 24h	199,83	254,58	422,91	241,29	1,77	1,90
Metformin 5 mM 24h	176,61	276,73	410,56	234,95	1,37	2,04
Metformin 5 mM 24h	194,98	226,09	388,62	194,06	1,42	2,11
4-IPP (α-MIF) 100 µM 24h	328,36	421,59	534,10	268,83	1,16	1,27
4-IPP (α-MIF) 100 µM 24h	303,01	389,42	548,27	239,66	1,25	1,25
4-IPP (α-MIF) 100 µM 24h	316,53	404,19	578,54	267,75	1,08	1,12
4-IPP (α-MIF) 100 µM 24h	323,30	436,20	553,44	266,23	1,75	1,45
4-IPP (α-MIF) 100 µM 24h	310,77	426,74	522,93	270,15	1,56	1,37

Suuplementary Table 3, page 2

Characteristics	7KC	27OHC	C14:1	C14	C16:1A
Untreated (DMSO) 24h	2,42	15,30	668,07	2960,37	631,17
Untreated (DMSO) 24h	2,96	15,20	597,15	2924,05	619,98
Untreated (DMSO) 24h	3,17	12,26	349,57	1828,27	496,53
Untreated (DMSO) 24h	3,19	14,88	525,31	2508,57	494,88
Untreated (DMSO) 24h	2,72	13,41	249,12	1297,01	431,54
Metformin 5 mM 24h	3,59	8,47	368,31	1764,62	405,73
Metformin 5 mM 24h	3,28	8,90	347,95	1714,14	361,58
Metformin 5 mM 24h	3,32	11,83	414,21	2164,56	421,02
Metformin 5 mM 24h	3,14	12,36	357,03	1977,79	407,14
Metformin 5 mM 24h	3,42	9,49	337,44	1716,97	387,80
4-IPP (α-MIF) 100 µM 24h	2,97	15,66	677,47	2596,00	574,64
4-IPP (α-MIF) 100 µM 24h	2,42	14,83	953,56	3512,51	513,55
4-IPP (α-MIF) 100 µM 24h	3,01	11,59	501,37	2223,28	561,82
4-IPP (α-MIF) 100 µM 24h	2,61	13,76	472,73	1991,31	556,12
4-IPP (α-MIF) 100 µM 24h	2,92	14,69	701,13	2753,62	632,09

Characteristics	C16:1B	C16	C18:1A	C18:1B	C18
Untreated (DMSO) 24h	62629,24	34928,12	77738,91	11327,57	27305,51
Untreated (DMSO) 24h	60934,29	32483,20	77033,89	12000,01	28097,90
Untreated (DMSO) 24h	38958,52	33467,24	71695,91	11236,41	25009,49
Untreated (DMSO) 24h	53709,23	32958,10	80596,86	11453,14	27063,59
Untreated (DMSO) 24h	27530,10	31974,36	71930,57	13050,64	25644,71
Metformin 5 mM 24h	34258,90	19826,23	50092,15	6481,53	16532,05
Metformin 5 mM 24h	36037,82	19717,26	49577,17	6202,27	16541,62
Metformin 5 mM 24h	38672,08	24186,78	51377,64	6352,32	18536,41
Metformin 5 mM 24h	33094,97	23940,66	59510,91	7574,91	18384,16
Metformin 5 mM 24h	33028,66	20051,28	51298,69	6945,06	16193,33
4-IPP (α-MIF) 100 µM 24h	50079,43	19889,63	59829,31	7420,20	20927,04
4-IPP (α-MIF) 100 µM 24h	37238,61	16175,61	56134,41	7461,97	19283,02
4-IPP (α-MIF) 100 µM 24h	41317,78	21951,68	55420,22	7025,17	20784,09
4-IPP (α-MIF) 100 µM 24h	42706,19	23121,01	56636,33	7017,04	21665,63
4-IPP (α-MIF) 100 µM 24h	55241,93	19324,86	55312,36	7340,32	20387,46

Suuplementary Table 3, pag 3

Characteristics	lactate	piruvate	glicerol 3P	succinate	fumarate	malate	citrate
Untreated (DMSO) 24h	3852,18	216,61	361,10	2506,33	406,01	908,53	586,46
Untreated (DMSO) 24h	3451,69	212,93	357,20	2202,30	365,99	819,17	438,60
Untreated (DMSO) 24h	3812,92	222,32	335,29	1900,77	348,41	855,15	581,49
Untreated (DMSO) 24h	3575,51	239,80	322,32	2055,85	372,61	959,64	454,45
Untreated (DMSO) 24h	3725,89	230,00	323,90	1926,57	378,29	855,45	546,87
Metformin 5 mM 24h	5747,40	207,56	420,97	565,07	300,93	666,10	147,95
Metformin 5 mM 24h	6292,29	191,27	419,22	552,84	279,78	674,66	135,45
Metformin 5 mM 24h	4543,52	191,26	435,49	659,09	311,94	652,22	157,45
Metformin 5 mM 24h	5086,87	225,24	453,99	641,60	319,59	593,86	185,19
Metformin 5 mM 24h	5553,04	214,18	456,87	607,31	267,40	562,26	168,30
4-IPP (α-MIF) 100 μM 24h	3052,36	259,14	556,06	336,94	111,67	233,16	126,92
4-IPP (α-MIF) 100 μM 24h	2614,66	240,14	603,31	306,00	119,30	272,54	127,98
4-IPP (α-MIF) 100 μM 24h	4186,57	247,95	503,79	434,13	90,91	254,19	126,87
4-IPP (α-MIF) 100 μM 24h	4225,18	234,52	568,87	353,03	82,85	254,05	119,70
4-IPP (α-MIF) 100 μM 24h	3771,29	241,68	607,61	397,41	109,69	319,58	120,54

Suuplementary Table 3, pag 4

	[ ] µg/µl	mgprot/vol	chol	latho	desmo	lano	7aOHC	7bOHC	7KC	27OHC
Mean unt	8,09	1,62	313,52	484,11	621,10	311,78	1,53	2,07	2,89	14,21
SD unt	2,29	0,46	5,79	40,10	35,50	3,81	0,23	0,06	0,32	1,33
CV% unt	28,30	28,30	1,85	8,28	5,72	1,22	15,13	3,00	11,19	9,37
Mean met	6,08	1,22	189,47	259,33	388,66	224,82	1,55	2,03	3,35	10,21
SD met	0,46	0,09	9,33	20,42	29,14	18,75	0,18	0,12	0,17	1,77
CV% met	7,64	7,64	4,92	7,87	7,50	8,34	11,44	5,84	4,99	17,29
Mean 4-IPP	2,85	0,57	316,39	415,63	547,46	262,52	1,36	1,29	2,79	14,11
SD 4-IPP	0,72	0,14	10,02	18,71	21,11	12,86	0,28	0,12	0,26	1,56
CV% 4-IPP	25,21	25,21	3,17	4,50	3,86	4,90	20,90	9,64	9,23	11,06
met/unt%	75,09	75,09	60,43	53,57	62,58	72,11	100,79	98,00	115,84	71,85
4-IPP/unt%	35,19	35,19	100,92	85,85	88,14	84,20	88,61	62,31	96,38	99,28

Date	[ ] µg/µl	mgprot/vol	C14:1	C14	C16:1A	C16:1B	C16	C18:1A	C18:1B	C18
Mean unt	8,09	1,62	477,85	2303,66	534,82	48752,27	33162,20	75799,23	11813,55	26624,24
SD unt	2,29	0,46	174,24	724,13	86,98	15098,36	1131,94	3876,46	752,38	1264,49
CV% unt	28,30	28,30	36,46	31,43	16,26	30,97	3,41	5,11	6,37	4,75
Mean met	6,08	1,22	364,99	1867,62	396,66	35018,49	21544,44	52371,31	6711,22	17237,52
SD met	0,46	0,09	29,77	198,22	22,88	2378,24	2304,57	4065,51	557,06	1126,30
CV% met	7,64	7,64	8,16	10,61	5,77	6,79	10,70	7,76	8,30	6,53
Mean 4-IPP	2,85	0,57	661,25	2615,34	567,65	45316,79	20092,56	56666,53	7252,94	20609,45
SD 4-IPP	0,72	0,14	192,62	584,71	42,70	7232,83	2673,99	1848,94	216,12	874,01
CV% 4-IPP	25,21	25,21	29,13	22,36	7,52	15,96	13,31	3,26	2,98	4,24
met/unt%	75,09	75,09	76,38	81,07	74,17	71,83	64,97	69,09	56,81	64,74
4-IPP/unt%	35,19	35,19	138,38	113,53	106,14	92,95	60,59	74,76	61,40	77,41

	[ ] µg/µl	mgprot/vol	Lactate	Pyruvate	Glycerol 3P	Succinate	Fumarate	Malate	Citrate
Mean unt	8,09	1,62	3683,64	224,33	339,96	2118,36	374,26	879,59	521,57
SD unt	2,29	0,46	167,63	10,78	18,27	247,79	21,00	54,94	70,41
CV% unt	28,30	28,30	4,55	4,81	5,37	11,70	5,61	6,25	13,50
Mean met	6,08	1,22	5444,62	205,90	437,31	605,18	295,93	629,82	158,87
SD met	0,46	0,09	663,69	14,78	17,74	46,33	21,88	49,21	19,05
CV% met	7,64	7,64	12,19	7,18	4,06	7,66	7,40	7,81	11,99
Mean 4-IPP	2,85	0,57	3570,01	244,69	567,93	365,50	102,89	266,70	124,40
SD 4-IPP	0,72	0,14	712,49	9,39	42,08	50,60	15,31	32,68	3,94
CV% 4-IPP	25,21	25,21	19,96	3,84	7,41	13,84	14,88	12,25	3,17
met/unt%	75,09	75,09	147,81	91,78	128,64	28,57	79,07	71,60	30,46
4-IPP/unt%	35,19	35,19	96,92	109,07	167,06	17,25	27,49	30,32	23,85

Supplementary Table 3.
4-IPP and Metformin treatments produce metabolic changes.
The table shows the values of the metabolites analyzed after treatment of C2 organoids with 100 µM 4-IPP at and 5 mM metformin.
The results were expressed as ng/mg of proteins and were normalized to the value of total proteins present in each sample.
All the experiments were performed in triplicate.
