# Supplementary material for: MIF/CD74 axis is a target for novel therapies in colon carcinomatosis
Source: J Exp Clin Cancer Res. 2017 Jan 23;36:16. doi: 10.1186/s13046-016-0475-z (PMC5260021; doi:10.1186/s13046-016-0475-z)
Supplement: Additional file 2: — Figure 1. Phase contrast microscope images of C1 and C2 organoids. Figure 2. Ingenuity Pathway Analysis of C2 secretome. Figure 3. Western blot analysis of C2 organoids after 4-IPP treatments. (PDF 282 kb) [file 13046_2016_475_MOESM2_ESM.pdf]

Supplementary Figure 1: Phase contrast microscope images of C1 and C2 organoids.

C1 C2

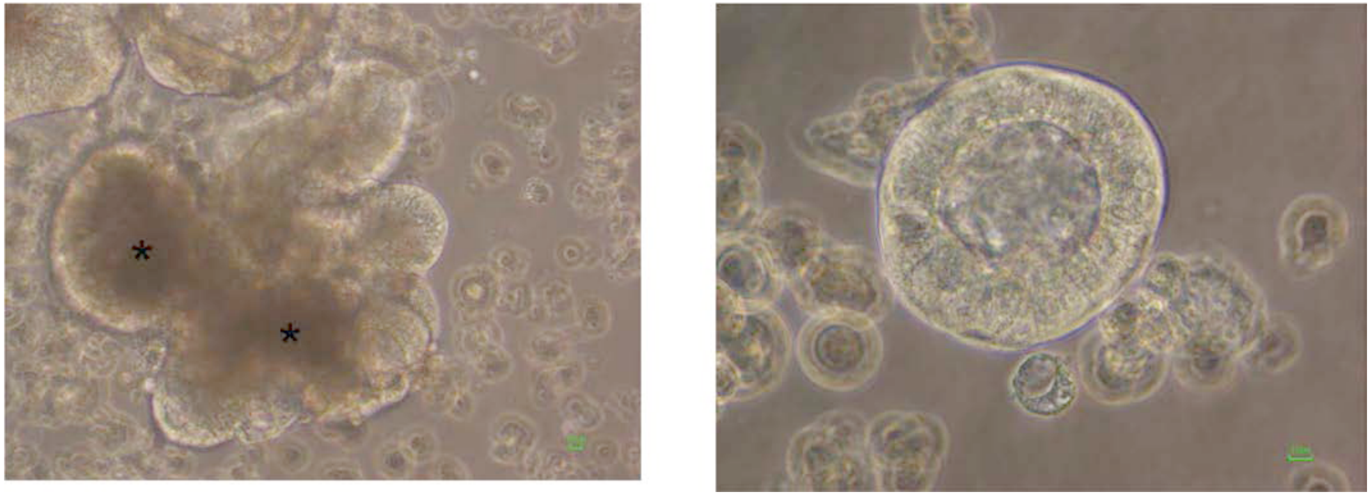

Supplementary Figure 2: Ingenuity Pathway Analysis of C2 Secretome

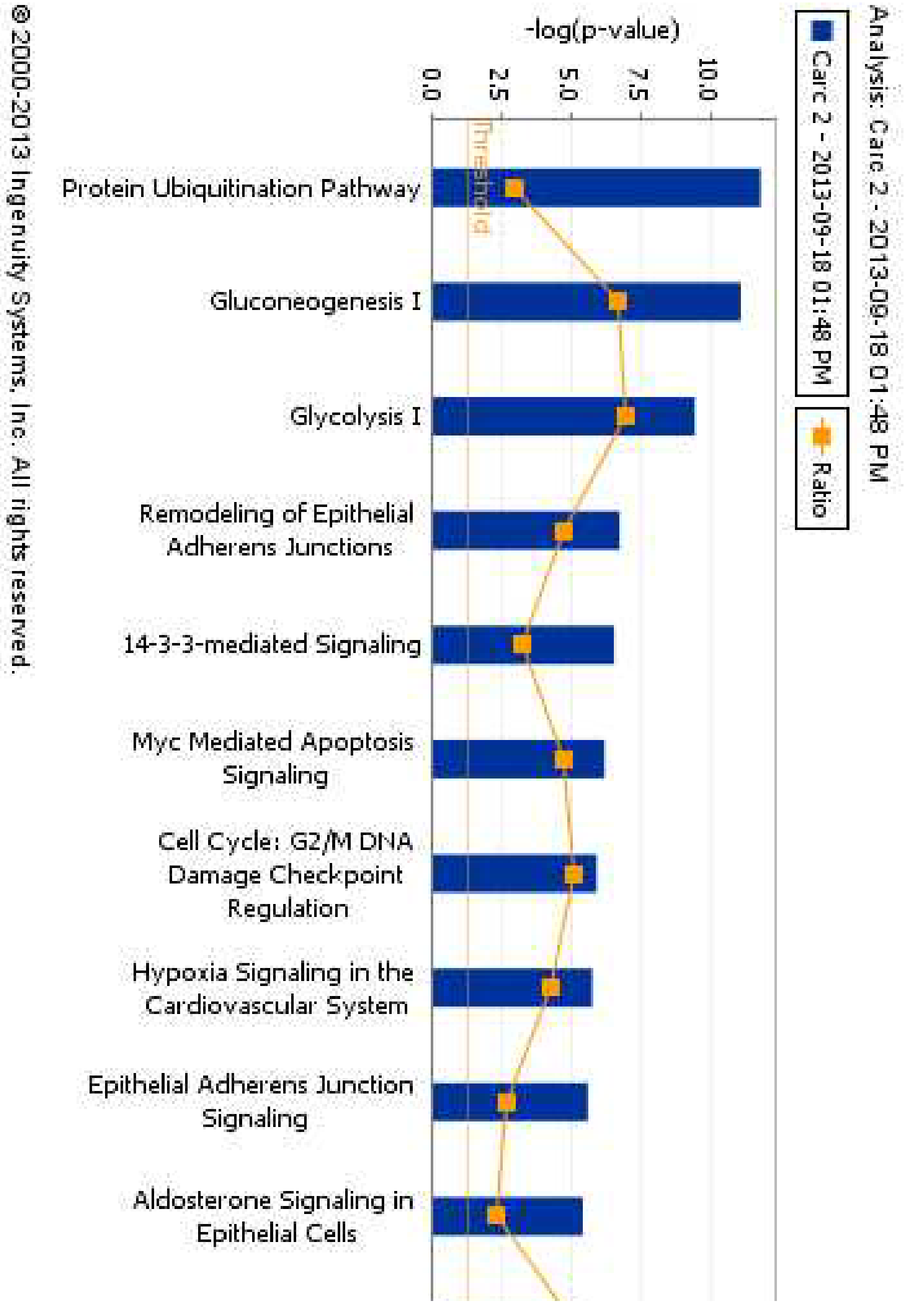

Supplementary Figure 3: Western blot analysis of C2 organoids after 4-IPP treatments.

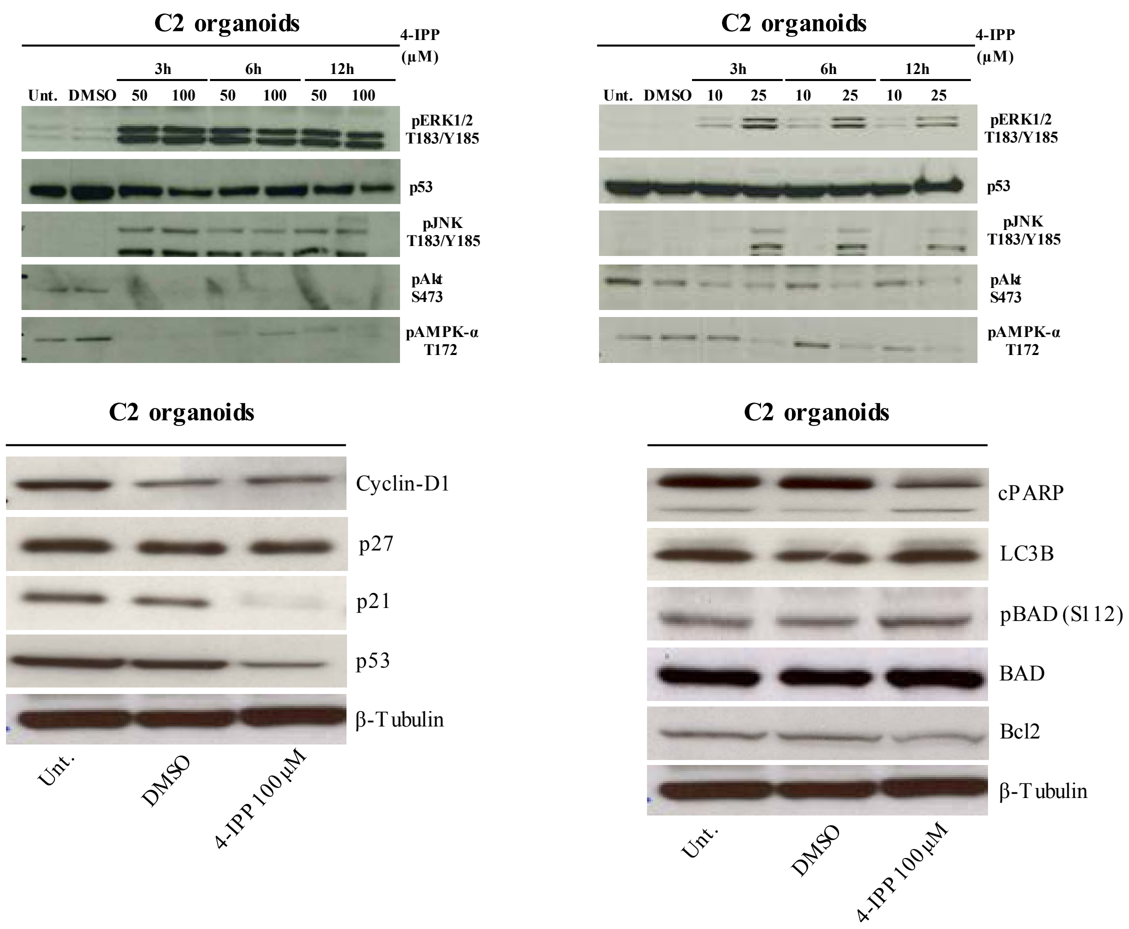

Supplementary Figure Legends

Figure 1.

By phase contrast microscope, C1 organoids showed both tubular-branched and more round shaped 3D structures. The central necrosis in C1 organoids is highligted by the asterisks C2 organoids were composed by round-shaped cellular aggregates.

Figure 2.

Top 10 canonical pathways derived from ingenuity pathway analysis (IPA) gene ontology algorithms for the 228 proteins derived from secretome of C2 organoids. These pathways emerged following IPA "Core Analysis." Graph shows category scores; "threshold" indicates the minimum significance level [scored as −log(p-value) from Fisher's exact test, set here to 1.25]. "Ratio" (differential yellow line and markers) refers to the number of molecules from the dataset that map to the pathway listed divided by the total number of molecules that map to the canonical pathway from within the IPA knowledgebase. The majority of secreted proteins are involved in protein turn over pathway and in metabolic functions (e.g., Protein ubiquitination Pathway, Gluconeogenesis 1 and glycolysis 1).

Figure 3.

Western blot analysis of pERK-1/2, pAkt, pJNK, pAMPK-α and p53 in C2 organoids with 4-IPP at 10, 25, 50 and 100 μM after 3, 6 and 12 h (above panels). Western blot analysis of Cyclin-D1, p27, p21, cPARP, LC3B, pBAD, BCL2 and p53 in C2 organoids with 4-IPP at 100 μM after 24 h (below panels). Normalization was obtained with immunoblot analysis of β-tubulin. Western blot analysis were performed at least in duplicate.
